# Environmental change drives accelerated adaptation through stimulated copy number variation

**DOI:** 10.1371/journal.pbio.2001333

**Published:** 2017-06-27

**Authors:** Ryan M. Hull, Cristina Cruz, Carmen V. Jack, Jonathan Houseley

**Affiliations:** Epigenetics Programme, The Babraham Institute, Cambridge, United Kingdom; New York University, United States of America

## Abstract

Copy number variation (CNV) is rife in eukaryotic genomes and has been implicated in many human disorders, particularly cancer, in which CNV promotes both tumorigenesis and chemotherapy resistance. CNVs are considered random mutations but often arise through replication defects; transcription can interfere with replication fork progression and stability, leading to increased mutation rates at highly transcribed loci. Here we investigate whether inducible promoters can stimulate CNV to yield reproducible, environment-specific genetic changes. We propose a general mechanism for environmentally-stimulated CNV and validate this mechanism for the emergence of copper resistance in budding yeast. By analysing a large cohort of individual cells, we directly demonstrate that CNV of the copper-resistance gene *CUP1* is stimulated by environmental copper. CNV stimulation accelerates the formation of novel alleles conferring enhanced copper resistance, such that copper exposure actively drives adaptation to copper-rich environments. Furthermore, quantification of CNV in individual cells reveals remarkable allele selectivity in the rate at which specific environments stimulate CNV. We define the key mechanistic elements underlying this selectivity, demonstrating that CNV is regulated by both promoter activity and acetylation of histone H3 lysine 56 (H3K56ac) and that H3K56ac is required for *CUP1* CNV and efficient copper adaptation. Stimulated CNV is not limited to high-copy *CUP1* repeat arrays, as we find that H3K56ac also regulates CNV in 3 copy arrays of *CUP1* or *SFA1* genes. The impact of transcription on DNA damage is well understood, but our research reveals that this apparently problematic association forms a pathway by which mutations can be directed to particular loci in particular environments and furthermore that this mutagenic process can be regulated through histone acetylation. Stimulated CNV therefore represents an unanticipated and remarkably controllable pathway facilitating organismal adaptation to new environments.

## Author summary

Evolutionary theory asserts that adaptive mutations, which improve cellular fitness in challenging environments, occur at random and cannot be controlled by the cell. The mutation mechanisms involved are of widespread importance, governing diverse processes from the acquisition of resistance during chemotherapy to the emergence of nonproductive clones during industrial fermentations. Here we ask whether eukaryotic cells are in fact capable of stimulating useful, adaptive mutations at environmentally relevant loci. We show that yeast cells exposed to copper stimulate copy number amplification of the copper resistance gene *CUP1*, leading to the rapid emergence of adapted clones, and that this stimulation depends on the highly regulated acetylation of histone H3 lysine 56. Stimulated copy number variation (CNV) operates at sites of preexisting copy number variation, which are common in eukaryotic genomes, and provides cells with a remarkable and unexpected ability to alter their own genome in response to the environment.

## Introduction

Copy number variation (CNV) is widespread in human populations, with 5%–10% of the human reference genome showing CNV between normal individuals [1–3]. CNV of protein-coding genes contributes to multiple disorders, and specific genetic syndromes have been directly attributed to CNV [4–6]. The pathological effects of CNV imply that gene copy number impacts gene expression, and we have recently shown that changing copy number can directly influence RNA processing [7]. However, CNV of protein coding genes is not always detrimental and can enhance cell growth, particularly in challenging environments. Evolution experiments in yeast give rise to novel CNVs that enhance growth under nutrient starvation, bestow drug resistance, and complement genetic defects [8–12]. CNVs in tumour cells also enhance proliferation, albeit at the expense of the host; for example, copy number amplification can drive tumour growth (e.g., of *FGFR2* or *CDK4* [13, 14]) or mediate drug resistance (e.g., of *DHFR*, *KRAS* or *BRAF* [15–17]). These yeast and human CNVs are examples of adaptive events in which the emergence of novel heritable alleles increases the reproductive fitness of the cell in the current environment.

The emergence of a novel allele in a population requires extensive selection such that the phenotypic observation is removed from original mutation event by many generations, and therefore causal mechanisms remain uncertain for most adaptive mutations including CNV [18]. Neo-Darwinian theory invokes natural selection of randomly occurring mutations to explain adaptation; however, random mutations need not be accidental, as the induction of genome-wide mutation under stress has been well characterised in bacteria and also reported in yeast [19–21]. Furthermore, a handful of loci in eukaryotes undergo localised and controlled genetic changes, including the mammalian immunoglobulin loci (widely reviewed, for example see [22–24]), as well as the budding yeast ribosomal DNA (rDNA) for which multiple CNV mechanisms have been described [25–27]. These loci are, however, highly specialised and their genetic changes are performed by locus-specific machinery; equivalent mechanisms acting genome-wide to induce beneficial genetic changes have not been convincingly demonstrated and present substantial theoretical difficulties [28–30].

**Fig 1:**
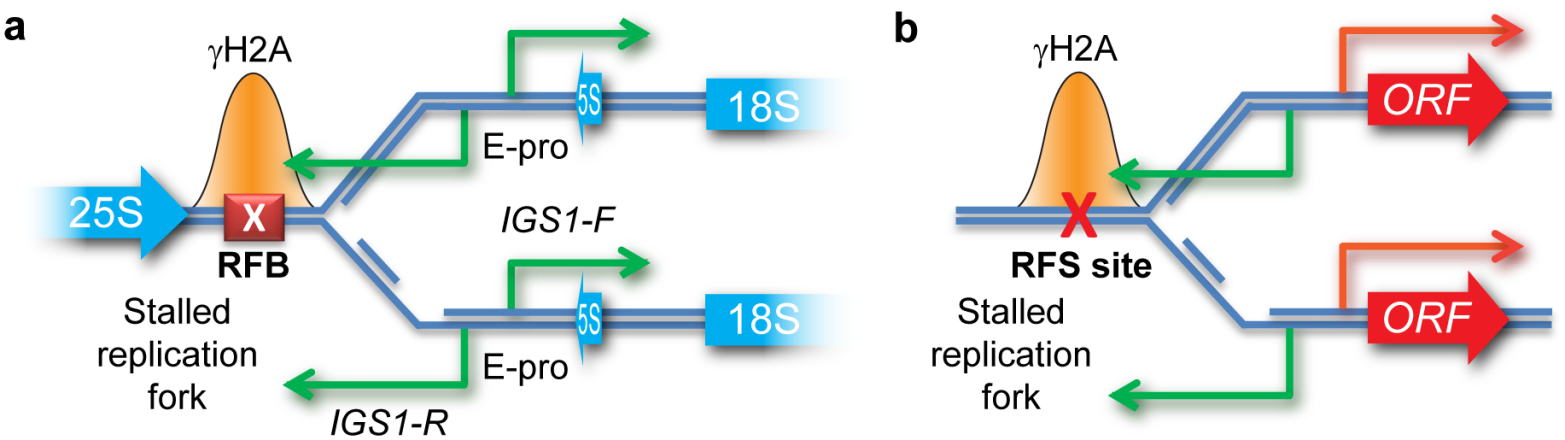
Systems for stimulated copy number variation (CNV) at the ribosomal DNA (rDNA) and at a model gene. **a**: Minimal elements implicated in control of rDNA recombination: transcription from bidirectional promoter E-pro and replication fork stalling at the Fob1-induced replication fork barrier (RFB). Green arrows represent noncoding RNAs *IGS1-R* and *IGS1-F* transcribed from the E-pro promoter; blue arrows show the rRNA genes (not to scale). **b**: Schematic representation of a general system in which a bidirectional promoter is adjacent to a replication fork stalling (RFS) site. Activation of the bidirectional promoter leads to transcription of the ORF (red arrow) and a noncoding RNA (green arrow). This system should, by analogy to the rDNA, be subject to stimulated CNV when the promoter for the indicated ORF is induced. Stalling of replication forks leads to an accumulation of S139-phosphorylated histone H2A (γH2A) (indicated by orange peaks) that can be detected by chromatin immunoprecipitation (ChIP).

The budding yeast rDNA has been used extensively as a model system for CNV. The rDNA consists of ~150 tandem copies of a 9.1-kb sequence encoding the ribosomal RNAs and undergoes frequent CNV [31]. rDNA recombination is initiated almost exclusively from a replication fork barrier (RFB) present in each rDNA copy (Fig 1a). A single protein, Fob1, defines the replication fork stalling site at the RFB [32, 33], and cleavage of these stalled forks is thought to initiate break-induced replication (BIR), a homologous recombination (HR) process that mediates replication fork restart using a homologous sequence on the sister chromatid [34]. Because homologous sequences are present in each rDNA copy, nonallelic homologous recombination (NAHR) occurs readily during BIR, causing frequent CNV. rDNA amplification is partly controlled through transcription; recombination requires RNA Pol I transcription [35], and NAHR is enhanced by expression of 2 noncoding RNAs (ncRNAs), *IGS1-F* and *IGS1-R* [27] (Fig 1a). *IGS1-F* is a stable ncRNA, whereas *IGS1-R* is a cryptic unstable transcript (CUT), a class of noncoding RNA that is degraded instantly after transcription and is transcribed through the RFB [36, 37]. Therefore, local transcription in the context of stalled replication forks at the RFB has a profound effect on rDNA CNV and is thought to cause the environmentally regulated rDNA amplification observed in cells with insufficient rDNA copy number [34, 38, 39].

Although the rDNA recombination machinery is locus specific, replication fork stalling is not unique to the RFB, and CNV often arises from replication defects [40–43]. Collisions between replication and transcription are known to be particularly mutagenic [44], and highly transcribed loci are prone to mutation in general [44–47]. Furthermore, transcription in bacteria has been directly observed to cause replisome dissociation, and mutation rates are higher for bacterial genes oriented against the direction of replication [48, 49]. This lead us to hypothesise that environmental nutrients and toxins that invoke strong transcriptional responses may promote mutations such as CNV at induced loci, effectively focusing mutations at genes that are important for growth in the presence of those nutrients or toxins, thereby accelerating the formation of novel alleles that confer increased fitness in the current environment. Here we demonstrate that CNV of high- and low-copy repeated sequences can be directly stimulated at induced loci in response to the environment, giving rise to novel, advantageous alleles at a rate far in excess of the basal mutation rate.

## Results

### Promoter induction can stimulate CNV

Replication fork stalling (RFS) occurs widely in the yeast genome and is generally mutagenic [42, 50–52]. By analogy to the rDNA system, we hypothesised that CNV may be instigated from RFS sites upstream of inducible promoters when those promoters are induced (Fig 1a and 1b). This would cause reproducible, environment-specific patterns of gene loss and gene amplification.

**Fig 2:**
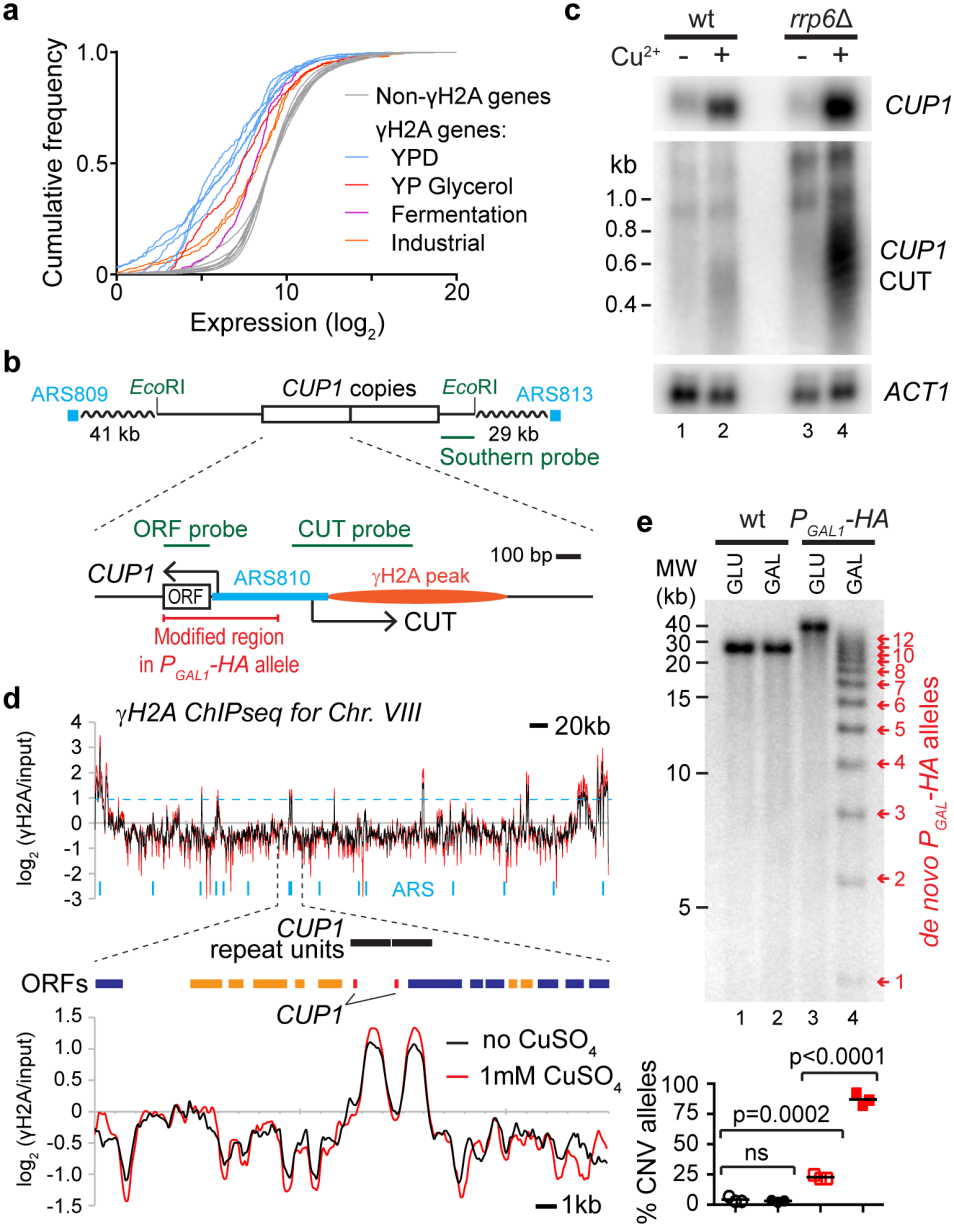
Candidate genes for stimulated copy number variation (CNV). **a**: Cumulative frequency distribution of gene expression for *S*. *cerevisiae* growing in various environments. Non-γH2A genes from all data sets are shown in grey, and γH2A genes are shown in blue for cells grown in YPD and in orange, red, and purple for other conditions (*p* = 0.00011, comparing γH2A genes in YPD to other conditions by nested ANOVA). **b**: Schematic of *CUP1* repeats and surrounding region of Chr. VIII, showing 2 copies of *CUP1* as annotated in the reference genome sequence (though the BY4741 wild-type [wt] used here actually has 13 copies). Close-up of a single *CUP1* copy is also shown. Probes used for northern and Southern blots are indicated in green, along with *Eco*RI sites used for Southern analysis. The nearest flanking replication origins (autonomously replicating sequences or ARS elements) are drawn in blue; each *CUP1* repeat also contains a putative ARS overlapping the *CUP1* promoter. The site of the γH2A peak in **d** is represented in orange. Arrows indicate transcription of *CUP1* mRNA and cryptic unstable transcript (CUT) from the *CUP1* promoter; the *CUP1* ORF is shown in white, and the region replaced by *P*_*GAL1*_*-HA* in the galactose-inducible construct is highlighted in red. **c**: Northern analysis of *CUP1* mRNA and *CUP1* upstream CUT in wild-type and *rrp6*Δ cells grown in YPD and exposed to 1 mM CuSO_4_ for 4 hours; *ACT1* is a loading control. **d**: ChIPseq data for γH2A in wild-type cells grown with or without 1 mM CuSO_4_, showing Chr. VIII and a close-up of the region surrounding the *CUP1* genes. The dotted blue line shows the cut off for peak calling, while blue vertical marks represent the annotated replication origins across the chromosome. **e**: Cells with *CUP1* ORF and promoter in each *CUP1* copy replaced by *P*_*GAL1*_*-HA*, grown in glucose or galactose for 10 generations compared to wild-type cells. DNA analysis by Southern blot; arrows indicate de novo alleles formed by CNV events, with numbers indicating *P*_*GAL*_*-HA* copy number. Copy numbers of parental alleles are 13 and 17 copies in the wild-type and the *P*_*GAL*_*-HA* strains, respectively. Quantification shows the percentage of alleles deviating from the parental copy number, *n* = 3; *p*-values calculated by 1-way ANOVA. ns, not significant. Raw quantitation data is available in S1, S3 and S5 Data.

RFS sites are marked by S139-phosphorylated histone H2A (γH2A) [42]. We used chromatin immunoprecipitation (ChIP) sequencing (ChIPseq) for γH2A to generate a high-resolution profile of RFS in the yeast genome, producing a map that is broadly in accord with published ChIP-microarray data [42]. This experiment showed that peaks of >2-fold γH2A enrichment occur within 1 kb upstream of ~7% of *Saccharomyces cerevisiae* genes, which we classed as γH2A genes (S1 Table). We then performed a meta-analysis of published RNA sequencing (RNAseq) data, comparing steady-state mRNA levels of γH2A and non-γH2A genes; this revealed that γH2A genes are expressed at unusually low levels in yeast grown under optimal culture conditions (30°C in YPD) (Fig 2a and S1a Fig, compare grey and blue lines). However, these γH2A genes are significantly induced under more challenging conditions, such as respiratory growth and industrial fermentation (Fig 2a and S1a Fig, red lines). γH2A genes are therefore biased towards those that are expressed primarily during growth in suboptimal conditions. However, these genes are not rapidly induced by osmotic or oxidative stress and are therefore not simply stress-response factors (S1b Fig). If the induction of RFS genes can instigate CNV, these CNV events should be more frequent at genes induced in response to specific environmental conditions.

To experimentally validate this prediction, we focused on 1 γH2A gene, *CUP1*, a well-studied gene encoding a metallothionein that sequesters excess copper [53, 54]. *CUP1* occurs in a tandem array of 2-kb repeats and has widely varying copy numbers amongst different yeast strains, with higher copy numbers conferring enhanced resistance to copper toxicity [55, 56]. The haploid strain BY4741 used here has a *CUP1* copy number of 13 in our assays, in keeping with previously reported estimates of *CUP1* copy number in the parental S288C background (10–15 copies); this copy number is high but by no means exceptional compared to wild isolates [57, 58]. As expected, most strains that we have tested from the BY4741-derived **a** mating–type deletion collection [59] also have 13 *CUP1* copies, while the S288C-derived MEP diploid has 2 *CUP1* alleles of 13 and 14 copies (see “Stimulated CNV accelerates the acquisition of copper resistance”).

*CUP1* is strongly induced by environmental copper, and we performed a northern blot analysis to determine whether the *CUP1* promoter is bidirectional like the rDNA ncRNA promoter, as promoter bidirectionality is important for rDNA CNV [27]. Bidirectional promoters are common in the yeast genome, but often the antisense RNA produced is an unstable CUT that is hard to detect in wild-type cells [36, 60]. We therefore analysed RNA from a wild type and from an *rrp6*Δ mutant that lacks a key exonuclease activity required for CUT degradation [36], revealing that the *CUP1* promoter is bidirectional, transcribing a CUT through the RFS site in response to copper exposure (Fig 2b and 2c). γH2A peaks upstream of the *CUP1* ORFs are readily seen in our γH2A ChIPseq data, and the *CUP1* RFS site is unaffected by growth in copper, in contrast to a previous report that ongoing transcription prevents RFS [42] (Fig 2d). This combination of an inducible bidirectional promoter adjacent to an RFS site fits our model derived from the rDNA locus (Fig 1b), making *CUP1* an excellent candidate for stimulated CNV, particularly as CuSO_4_ induces only a handful of other γH2A genes (S2 Fig).

Copper exposure leads to the emergence of cells carrying amplified *CUP1* alleles [61], but proving that the environment actually stimulates *CUP1* CNV requires the measurement of CNV in the absence of selective pressure. To achieve this, we reengineered the *CUP1* repeat sequence, replacing the copper-responsive *CUP1* promoter and ORF with a *GAL1* promoter and a *3*x*HA* tag ORF while leaving surrounding sequences, including the RFS region, intact (Fig 2b). This modified construct expresses a nonfunctional protein in response to environmental galactose but not glucose, whereas the endogenous promoter is not induced by galactose. We inserted a construct containing 3 copies of this modified *P*_*GAL1*_*-HA* repeat in place of the *CUP1* locus, which fortuitously amplified to 17 copies upon transformation. *P*_*GAL1*_*-HA* cells were then grown for 10 generations in glucose or galactose and compared to wild-type controls grown under the same conditions. Growth of the *P*_*GAL1*_*-HA* strain in galactose gave rise to multiple de novo CNV alleles, detected by Southern blot, whereas no change was observed in the wild-type controls (Fig 2e). This demonstrates that promoter induction in the genetic context of *CUP1* is sufficient to stimulate CNV.

### Stimulated CNV accelerates the acquisition of copper resistance

Equivalent experiments, however, on the wild-type *CUP1* locus would not be informative because growth in the presence of copper selects for rare amplified alleles whether they arise through random or stimulated CNV. To determine whether copper stimulates *CUP1* CNV requires the analysis of many individual cells that are allowed to replicate with or without copper while excluding cells born during the exposure period. Quantification of de novo CNV events within this defined cohort would provide a measure of CNV rate independent of selection.

**Fig 3:**
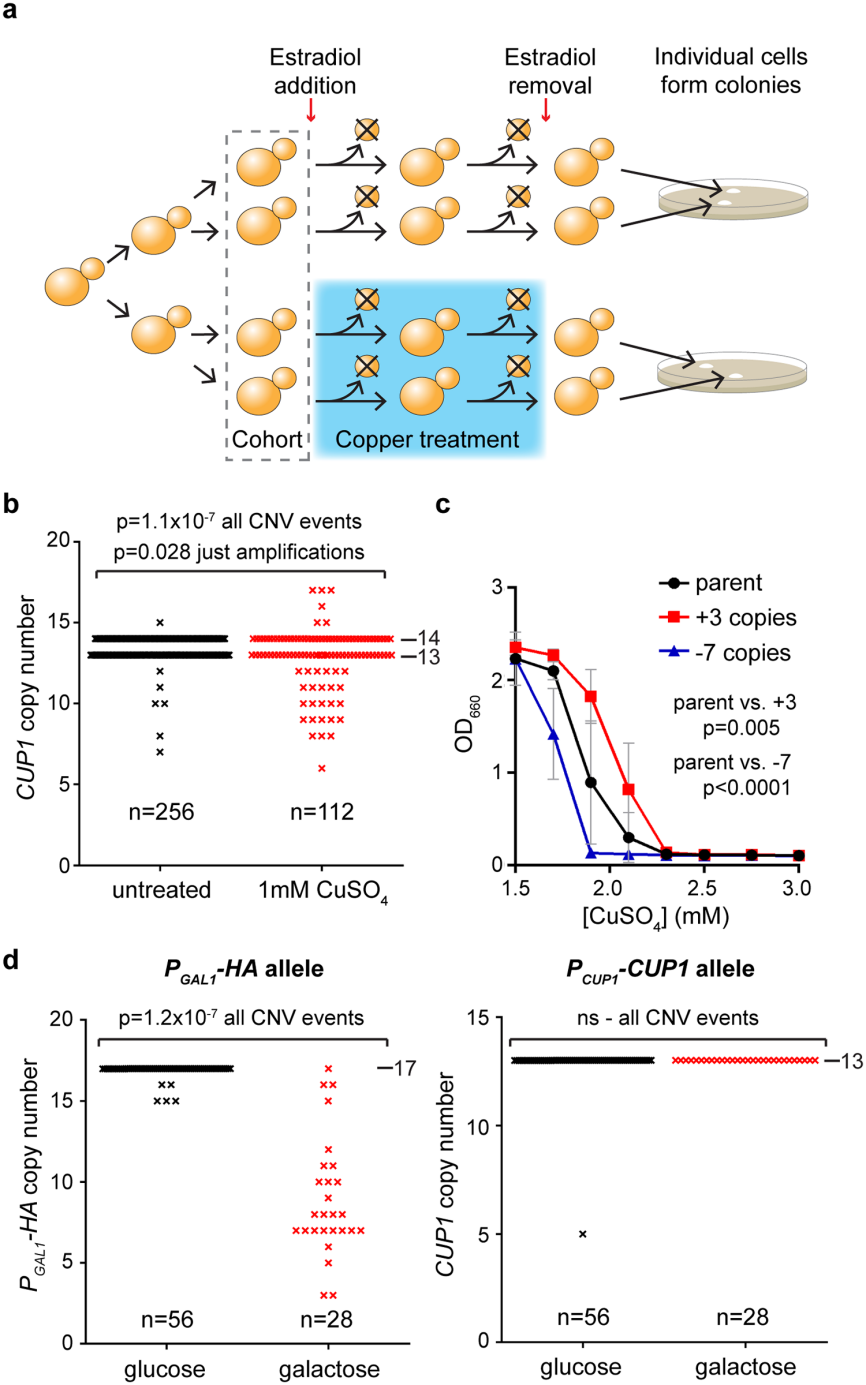
Stimulated copy number variation (CNV) in copper-treated cells. **a**: Strategy for quantifying stimulated CNV. Schematic of experimental system for measuring CNV in a defined cohort of mother enrichment program (MEP) cells. **b**: Copy number of *CUP1* alleles in colonies derived from 184 diploid MEP cells (pooled from 2 experiments), treated with or without 1 mM CuSO_4_ for 24 hours (128 cells for–Cu, 56 cells for +Cu). 89% of starting cohort were recovered in the untreated cohort, and 40% were recovered in the treated cohort. Observed mutation rates in the untreated cohort were normalised for the viability in the treated cohort, making the conservative assumption that cells lost during the experiment did not undergo CNV. *p*-values were calculated by a goodness of fit χ^2^ test with 1 degree of freedom between the observed and expected number of mutations to wild-type alleles across the cohorts. **c**: Copper resistance of 3 colonies recovered in **b** with parental, +3, and –7 *CUP1* copy numbers on 1 allele; CuSO_4_ was added at indicated concentrations to media containing 0.5 mM ascorbic acid to increase copper toxicity, and OD_660_ was measured after 3 days at 30°C. Error bars represent ±1 SD; *p*-values were calculated by 1-way ANOVA of area under curves; *n* = 6 for each group. **d**: Experiment as in **b** using heterozygous diploid cells with 1 wild-type *CUP1* allele and 1 *P*_*GAL1*_*-HA* allele; data are shown for both alleles in the same cells. Allele-specific probes covering the *CUP1* promoter and ORF or the *GAL1* promoter and *HA* ORF were used for this experiment. Copy numbers of parental alleles are indicated on each panel. ns, not significant. Raw quantitation data are available in S6 Data.

To achieve this, we employed the mother enrichment program (MEP)—a system that selectively renders new-born cells inviable in the presence of β-estradiol [62]. A cohort of MEP cells in the presence of β-estradiol can be treated for a given period with or without copper, after which time only cells in the initial cohort are viable (Fig 3a). Cells from copper-treated and control cohorts are then plated in the absence of copper and estradiol, giving rise to colonies derived from single cells that have (or have not) been previously exposed to copper; these colonies inherit the *CUP1* copy number of the progenitor cell. If copper stimulates heritable CNV at *CUP1*, then a greater number of colonies with *CUP1* alleles that deviate from the parental copy number should be detected in the copper-treated cohort.

We divided 2 populations of β-estradiol–treated MEP diploid cells and grew them for 24 hours in the presence or absence of 1 mM CuSO_4_, then assayed 184 of the resulting colonies for *CUP1* copy number (Fig 3b). We observed 31 CNV events (including 6 amplifications) in 56 copper-treated diploid cells (112 *CUP1* alleles, 27% CNV events, 5% amplifications), compared to 7 CNV events (including 1 amplification) in 128 untreated cells (256 alleles, 3% CNV events, 0% amplifications). The difference in the number of CNV events and amplifications between copper-treated and untreated cells is significant (*p* = 1.1x10^-7^ for CNV events and *p* = 0.028 for amplifications) and represents a 9-fold stimulation of CNV by copper. Furthermore, based on bud scar counting, the untreated cells undergo more divisions than the copper-treated cells in 24 hours (12 ± 2 versus 8 ± 3 divisions), meaning that 9-fold is an underestimate of the true extent of CNV stimulation. This finding directly demonstrates that environmental copper stimulates CNV at *CUP1*.

Changes in *CUP1* copy number alter copper resistance, and we therefore measured the ability of cells arising in this experiment bearing an amplified (+3) or a contracted (–7) allele to grow at different copper concentrations. As expected, copper resistance was significantly increased in the amplified clone and decreased in the contracted clone (Fig 3c). This demonstrates that stimulated CNV gives rise to de novo alleles with quantifiable phenotypic differences, including increased copper resistance.

To ensure that stimulated CNV is a specific result of promoter induction as opposed to a mutagenic effect of copper treatment, we created diploid MEP cells heterozygous for the wild-type *CUP1* allele and the engineered galactose-responsive *P*_*GAL1*_*-HA* allele. As predicted, galactose treatment induced extensive CNV at the P_*GAL1*_*-HA* allele (96% of *P*_*GAL1*_*-3HA* alleles underwent CNV in galactose compared to 9% in glucose) (Fig 3d, left). In contrast, the copper-responsive wild-type allele in the same cells was unaffected (0% of alleles underwent CNV in galactose and only 2% in glucose) (Fig 3d, right). This confirms that CNV is not stimulated uniformly and is highly selective for a transcriptionally induced allele over a silent locus of similar sequence and copy number.

### H3K56 acetylation is required for stimulated CNV

**Fig 4:**
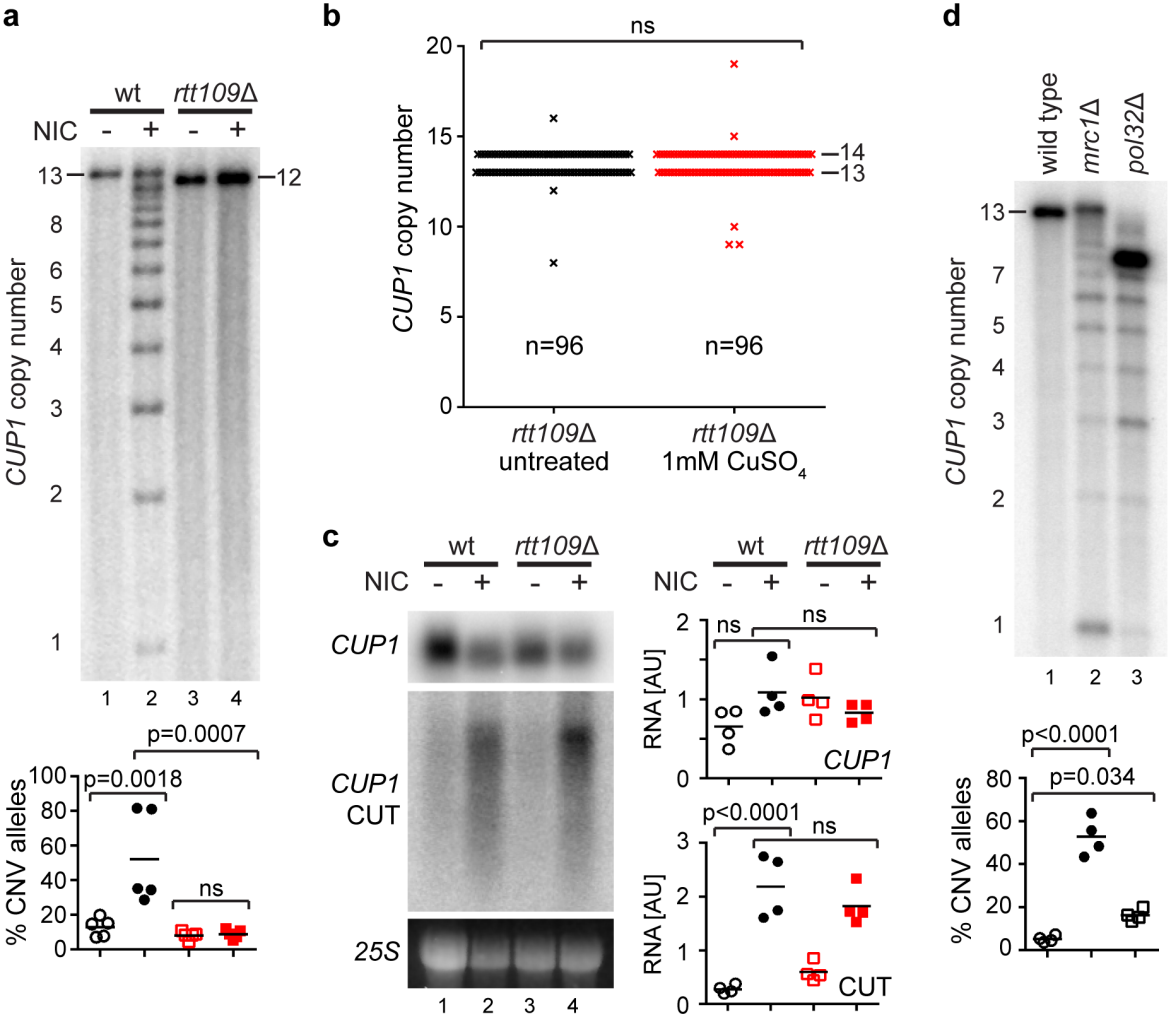
H3K56 acetylation has a critical role in stimulated copy number variation (CNV). **a**: Southern analysis of *CUP1* copy number in wild-type (wt) and *rtt109*Δ cells with 13 *CUP1* copies grown for 10 generations with or without 5 mM nicotinamide (NIC). Quantification shows the percentage of alleles deviating from the parental copy number after 10 generations; *n* = 5, *p*-values calculated by 1-way ANOVA. **b**: Measurement of CNV in a defined cohort of MEP *rtt109*Δ cells, performed exactly as Fig 3b; 48 diploid cells per condition. ns, not significant. **c**: Northern analysis of *CUP1* ORF and *CUP1* cryptic unstable transcript (CUT) RNA in log-phase wild-type and *rtt109*Δ cells with or without 5 mM nicotinamide. Quantification shows relative RNA levels in arbitrary units (AUs); *n* = 4, *p*-values calculated by 1-way ANOVA. **d**: Southern analysis of *mrc1*Δ and *pol32*Δ cells as in **a**, *n* = 4. Copy numbers of parental alleles are indicated on each panel. Note that in **d**, the mutants are derived from the wild type and therefore have a parental copy number of ~13, even though this allele is no longer detectable in the *pol32*Δ mutant. Raw quantitation data are available in S3, S4 and S6 Data.

Sir2 family histone deacetylases (HDACs) repress rDNA CNV at multiple levels, leading us to question whether HDACs also control *CUP1* CNV [26, 63]. Indeed, we observed extensive *CUP1* CNV after treating wild-type cells with the Sir2 family inhibitor nicotinamide (Fig 4a). Analysis of individual deletion mutants revealed that Sir2 itself has little impact on CNV at *CUP1*, but loss of the degenerate H3K56 HDACs Hst3 and Hst4 induces extensive CNV, suggesting a critical role for H3K56ac in regulating *CUP1* copy number (S3a Fig). Consistent with this, loss of the H3K56 acetyltransferase Rtt109 rendered the *CUP1* locus immune to nicotinamide (Fig 4a).

To determine the importance of Rtt109 for stimulated CNV, we repeated the MEP-based assay from Fig 3b in an *rtt109*Δ background. Remarkably, we found that the transcriptional stimulation of CNV in response to copper was completed abrogated by loss of Rtt109 (compare Fig 4b [showing *rtt109*Δ cells] to Fig 3b [showing wild-type cells]). This shows that stimulated CNV acts by a defined mechanism involving H3K56ac.

H3K56ac has been implicated in both *CUP1* promoter induction [64] and replication fork stability or restart [65–67]. Nicotinamide may therefore affect the stimulated CNV mechanism in 2 ways: by inducing the *CUP1* promoter or by destabilising the stalled replication fork. We observed that nicotinamide stimulates expression of the *CUP1* antisense CUT but causes little or no change in the level of the *CUP1* sense mRNA (Fig 4c). This suggests that HDAC inhibition by nicotinamide reduces promoter directionality at *CUP1* rather than inducing the promoter per se, as has been recently reported for other (as yet unidentified) HDACs [68]. This loss of directionality cannot be ascribed to H3K56 acetylation as it is also observed in *rtt109*Δ cells, and it must depend on another member of the nicotinamide-sensitive Sir2 family. Importantly, however, loss of promoter directionality cannot be solely responsible for *CUP1* CNV, as an equivalent increase in *CUP1* CUT transcript is observed in *rtt109*Δ cells in which CNV does not occur (compare Fig 4a to 4c). Therefore, nicotinamide treatment makes the *CUP1* promoter transcribe bidirectionally, but this effect is not Rtt109-dependent and is not the sole driver of CNV stimulation.

To assess the potential impact of H3K56 acetylation–associated replication fork defects on CNV, we asked whether mutations that destabilise or impair the processing of stalled replication forks phenocopy nicotinamide without affecting *CUP1* promoter induction or directionality. Amongst 11 deletion mutants of replication fork–associated proteins that impact rDNA stability, we observed that *mrc1*Δ and *pol32*Δ cells undergo striking *CUP1* CNV without affecting the *CUP1* promoter (Fig 4d and S3b Fig). Mrc1 stabilises stalled replication forks [69], while Pol32 is required for efficient DNA synthesis following the BIR events that are initiated from broken replication forks [70, 71]. The high level of *CUP1* CNV observed in both mutants is consistent with abnormally frequent or inefficient BIR being a key driver of CNV. Importantly, increased H3K56ac was recently shown to impair DNA synthesis during BIR, causing frequent replication fork stalling and recombination events [72]. Such additional recombination events occurring in a repetitive region should cause extensive CNV, providing a simple explanation for the induction of *CUP1* CNV by nicotinamide, which increases H3K56ac globally through inhibition of Hst3 and Hst4.

### Regulation of stimulated CNV by promoter activity and H3K56ac

**Fig 5:**
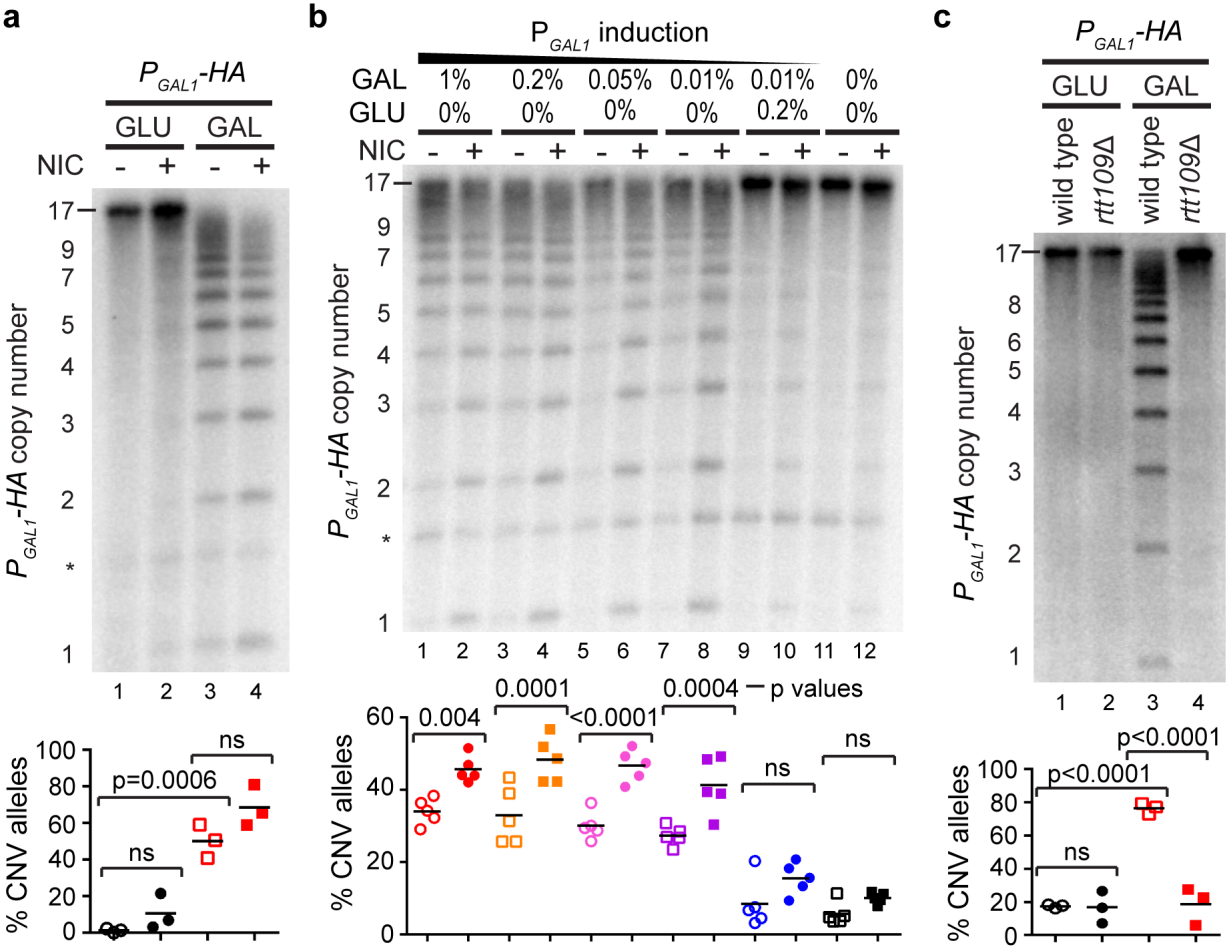
Combinatorial action of promoter activity and histone H3 lysine 56 acetylation (H3K56ac) on copy number variation (CNV). **a**: Southern analysis of copy number for *P*_*GAL1*_*-HA* cells grown for 10 generations in glucose (GLU) and galactose (GAL) with or without 5 mM nicotinamide (NIC). Quantification shows the percentage of alleles deviating from the parental copy number after 10 generations; *n* = 3, *p*-values calculated by 1-way ANOVA. * nonspecific band. ns, not significant. **b**: Southern analysis as in **a** using given concentrations of galactose and glucose combined with 2% raffinose. *n* = 5. * nonspecific band. *p*-values were calculated from pairwise comparisons of samples with or without NIC for each GLU or GAL concentration deriving from a 1-way ANOVA of the whole data set. See also alternative analysis in S5 Fig. **c**: Southern analysis as in **a** using wild-type or *rtt109*Δ derivatives of *P*_*GAL1*_*-HA* cells. *n* = 3. Copy numbers of parental alleles are indicated on each panel. Raw quantitation data are available in S3 Data.

To our surprise, however, nicotinamide had little effect on the *P*_*GAL1*_*-HA* allele, showing that a global increase in H3K56 acetylation is not sufficient to drive CNV (Fig 5a). One difference between the wild-type *CUP1* allele and the re-engineered *P*_*GAL1*_*-HA* allele is that the *GAL1* promoter is fully repressed in glucose, and therefore nicotinamide treatment does not cause the expression of an antisense transcript (S4 Fig). Given the dual effect of nicotinamide on *CUP1* promoter directionality and post-BIR replication, we suspected that both activities might be required for efficient CNV induction.

To test this, we grew *P*_*GAL1*_*-HA* cells with or without nicotinamide in a range of galactose concentrations known to induce the bidirectional *GAL1* promoter to various extents [73]. As predicted, the effect of nicotinamide was minimal and not significant without promoter induction (Fig 5b, lanes 9–12), but with higher promoter induction, nicotinamide significantly stimulated CNV (Fig 5b, lanes 1–8). This effect was particularly striking for the formation of de novo alleles with 1–3 copies, which presumably arise through multiple sequential CNV events (S5 Fig). This experiment reveals that promoter activity and H3K56ac make additive contributions to CNV.

However, we observed that CNV becomes largely independent of nicotinamide at high galactose concentrations (Fig 5a, lanes 3–4), which is not consistent with the model proposed above whereby H3K56ac acts during BIR. We therefore tested the importance of H3K56ac in CNV induction from the *GAL1* promoter by deleting *RTT109*. Just as for the wild-type *CUP1* locus, this completely abrogated CNV induction, confirming that H3K56ac is critical for stimulated CNV in the *P*_*GAL1*_*-HA* system (Fig 5c). These data show that stimulated CNV requires transcription and H3K56ac, but for highly induced promoters the normal physiological level of H3K56ac is sufficient to support extensive CNV such that further deregulation of H3K56 HDAC activity has little effect.

### Evidence for stimulated CNV in low-copy repeats

Direct detection of stimulated CNV is facilitated by the high copy number of the *CUP1* locus. However, this raises the question of whether CNV stimulation is restricted to high-copy tandem repeat loci, which are rare amongst protein-coding genes. In contrast, copy numbers of 2–5 are very common in the yeast and human genome sequences [1, 14, 74–77], and we asked whether a 3-copy *CUP1* locus would show equivalent behaviour to the high-copy system. Individual CNV events are too rare in this 3-copy system for direct detection but, having defined the effects of modulating H3K56 acetylation on CNV, we reasoned that if stimulated CNV acts at low-copy loci then H3K56 modulation should alter the rate at which *CUP1* amplifications emerge under copper selection in a predictable manner.

**Fig 6:**
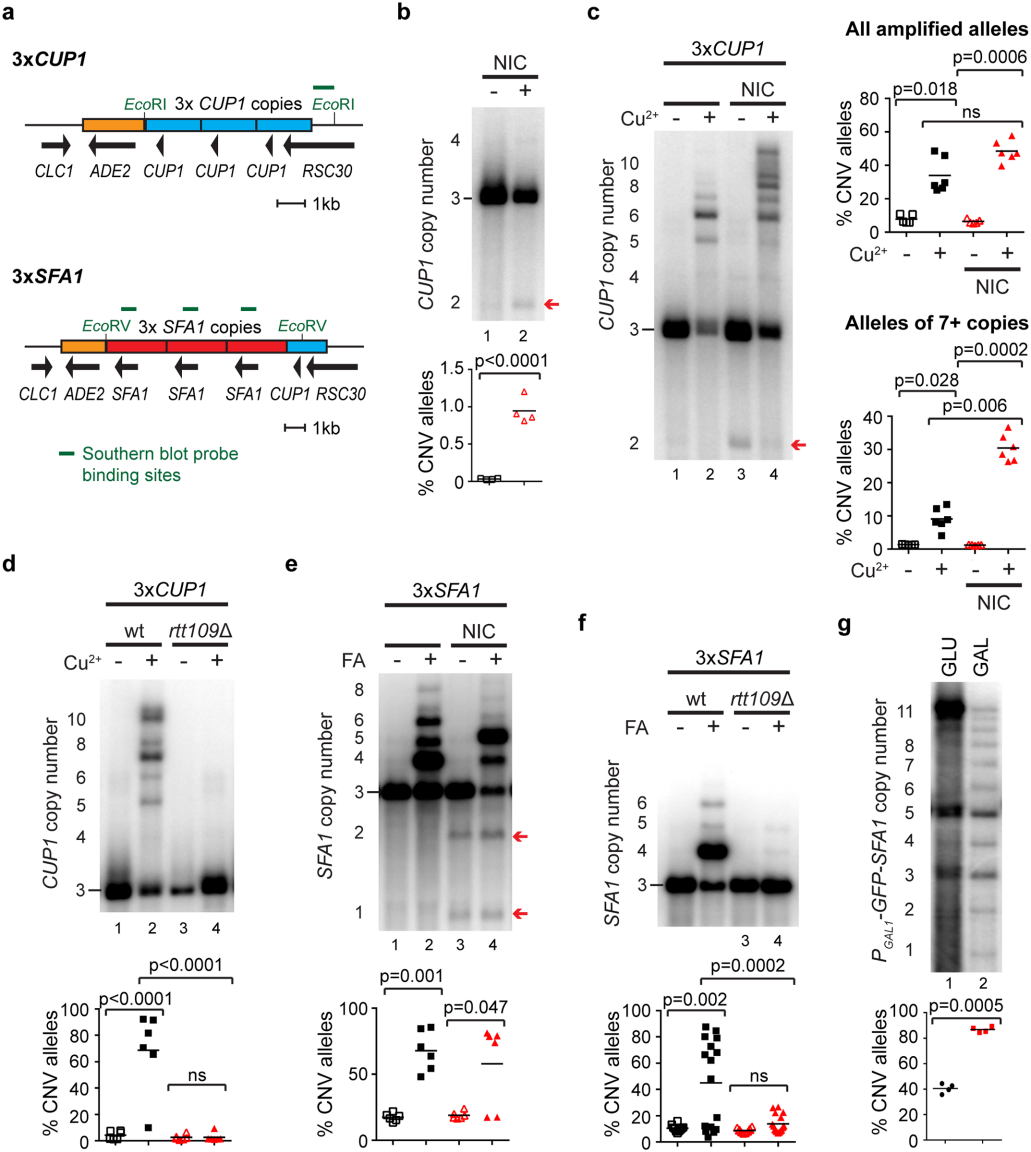
Stimulated copy number variation (CNV) in low-copy repeat systems. **a**: Schematics of the 3x*CUP1* and 3x*SFA1* constructs inserted at the endogenous *CUP1* locus. Blue boxes indicate *CUP1* repeats, red boxes indicate *SFA1* repeats, and orange boxes indicate the *ADE2* marker. The reading frame of *RSC30* is maintained across the construct boundary. Restriction enzymes for Southern analysis are shown in green along with probe locations. **b**: Southern analysis of *CUP1* copy number in 3x*CUP1* cells grown for 10 generations with or without 5 mM nicotinamide (NIC); arrow indicates –1 copy band. Quantification shows the percentage of –1 alleles; *n* = 4, *p*-value calculated by *t* test. **c**: Southern analysis of *CUP1* copy number in 3x*CUP1* cells grown for 10 generations with or without 0.3 mM CuSO_4_ and with or without 5 mM NIC. Upper quantification shows the percentage of alleles deviating from the parental copy number; *n* = 5, *p*-values calculated by 1-way ANOVA for repeated measurements. Lower quantification is as upper quantification, considering only alleles of 7+ copies. ns, not significant. **d**: Southern analysis of *CUP1* copy number in 3x*CUP1* wild-type (wt) and *rtt109*Δ cells after 10 generations with or without 0.3 mM CuSO_4_ (analysis as in **c**); *n* = 6. **e**: Southern analysis of *SFA1* copy number in 3x*SFA1* grown for 17 generations with or without ~1 mM formaldehyde (FA) and with or without 5 mM NIC. Quantification shows the percentage of alleles deviating from the parental copy number; *n* = 6, *p*-values calculated by 1-way ANOVA. A correction was applied to the quantification to account for the differing number of probe-binding sites in the amplified alleles. **f**: Southern analysis of *SFA1* copy number in 3x*SFA1* wild-type and *rtt109*Δ cells grown for 17 generations with or without ~1 mM FA. Quantification as in **e**; *n* = 10 for untreated samples, *n* = 16 for FA-treated samples. **g**: Southern analysis of CNV induced in the high-copy P_*GAL1*_-*GFP-SFA1* system. After selection for high copy number and outgrowth in SC media without FA, cells were grown for 10 generations in SC with 2% glucose (GLU) or galactose (GAL). Quantification shows the percentage of contracted alleles; *n* = 4, *p*-value calculated by paired *t* test. Raw quantitation data are available in S3 Data.

To test this, we replaced the endogenous *CUP1* locus with a synthetic construct containing 3 wild-type copies of the *CUP1* repeat sequence while maintaining the reading frame of the overlapping *RSC30* gene (Fig 6a). Stimulated CNV is replication linked, effectively requiring cells to grow in a sublethal concentration of copper. We observed that 3x*CUP1* cells grow slowly in 0.3 mM CuSO_4_, although fast-growing resistant populations often emerge late in growth, showing that resistant cells are under positive selection (S6a Fig). Importantly, when 3x*CUP1* cells were grown for 10 generations in batch culture in the presence of 0.3 mM CuSO_4_, copy-number-amplified alleles were almost always detected in the population by Southern blot (S6b Fig), forming a quantitative assay for CNV.

We first tested whether nicotinamide treatment stimulated CNV in 3x*CUP1* cells as in the high-copy system. Nicotinamide largely stimulates contractions in high-copy *CUP1* arrays, and in 3x*CUP1* cells the only reproducible CNV event observed on nicotinamide treatment was a –1 contraction to 2x*CUP1* (Fig 6b, red arrow). In combination with 0.3mM CuSO_4_, the 2x*CUP1* band largely disappeared, as would be expected under copper selection (Fig 6c, red arrow), and the proportion of amplified alleles increased marginally (Fig 6c, upper quantification panel). However, the most noticeable difference was that the proportion of large alleles (more than 2-fold the progenitor allele size) increased dramatically with nicotinamide treatment (Fig 6c, lower quantification panel). These results show that copper and nicotinamide both stimulate CNV, and although CNV stimulation causes many copy number contractions, copper and nicotinamide have an additive effect in the 3x*CUP1* system that results in the formation of larger alleles that predominate in batch culture.

In contrast, since deletion of *RTT109* suppressed stimulated *CUP1* CNV in the high-copy system, we then asked if the amplifications observed in 3x*CUP1* cells are also Rtt109-dependent. Indeed, when 3x*CUP1 rtt109*Δ cells were grown for 10 generations in 0.3 mM copper, amplification was completely suppressed (Fig 6d). This demonstrates that H3K56 acetylation is required for *CUP1* amplification in the presence of copper, a very surprising result because previous studies have shown a critical role for Rtt109 in maintaining genome stability rather than promoting genome change [26, 66, 78, 79].

We then asked whether another 3-copy RFS gene would show similar behaviour. We selected to test the *SFA1* gene encoding a formaldehyde dehydrogenase, as this has a clear upstream RFS site (S6c Fig), is inducible in response to formaldehyde, and higher *SFA1* copy number increases formaldehyde resistance [80]. A tandem array of 3 *SFA1* genes with surrounding sequence was inserted at the *CUP1* locus along with a single wild-type *CUP1* copy (Fig 6a), while the endogenous *SFA1* gene was deleted. These 3x*SFA1* cells showed a sharp cutoff for growth in formaldehyde, with <0.9 mM allowing robust growth and >1 mM completely suppressing growth, while the formaldehyde concentration that gave slow but reproducible growth (which is required for these assays) varied from 0.9–1.0 mM with formaldehyde batch and had to be empirically determined. We therefore refer to the assay concentration as ~1 mM formaldehyde.

Growth of 3x*SFA1* cells with ~1 mM formaldehyde induced bidirectional transcription from the *SFA1* promoter and again gave rise to amplified *SFA1* alleles detectable by Southern blot over 17 generations (Fig 6f and S6d Fig). As in the 3x*CUP1* system, growth of 3x*SFA1* cells in nicotinamide induced copy number contractions that were readily detected in the absence of formaldehyde (Fig 6e, red arrows), although the additive effect between formaldehyde and nicotinamide was not observed. Importantly however, the copy number amplification of *SFA1* in 3x*SFA1* cells was completely suppressed in an *rtt109*Δ mutant (Fig 6f), indicating that *SFA1* amplification proceeds in the presence of formaldehyde by the same mechanism as *CUP1* amplification.

Confirming that CNV is transcriptionally stimulated at *SFA1* requires a high-copy system, but we were unable to create a direct equivalent of the high-copy *P*_*GAL1*_*-HA* strain as this amplified fortuitously during transformation. The alternative is to select for an amplified allele using formaldehyde; however, this requires the *SFA1* gene and amplification system to be active, which is not the case if the *SFA1* promoter is simply replaced with *P*_*GAL1*_. We suspected that bidirectional transcription into the *SFA1* RFS would stimulate CNV irrespective of where the promoter is placed, so we created a construct in which the promoter and ORF of the upstream divergent gene *UGX2* were replaced with *P*_*GAL1*_*-GFP* in each of the 3 *SFA1* repeats (S6e Fig). This strain was grown in 0.9 mM formaldehyde, stepped up to 2.2 mM formaldehyde and then recovered in glucose media, yielding a *P*_*GAL1*_*-GFP-SFA1* strain with an unstable and therefore somewhat heterogeneous copy number but primarily containing 11 copies. Growth of this strain in galactose caused the disappearance of this upper band and the emergence of a prominent ladder (Fig 6g), just as in the original *P*_*GAL*_*-HA* strain (Fig 2e), showing that transcription directly stimulates CNV at the *SFA1* RFS site.

Together, these data show that low-copy systems undergo CNV through a mechanism consistent with stimulated CNV, and that this mechanism is not restricted to *CUP1*.

### Stimulated CNV enhances adaptation in a population

The ability of transcription to stimulate CNV and the reliance of this mechanism on H3K56 acetylation suggest that the emergence of copper adaptation, instead of being an inevitable consequence of random mutation, follows a defined mechanism that is highly sensitive to alterations in this histone mark.

**Fig 7:**
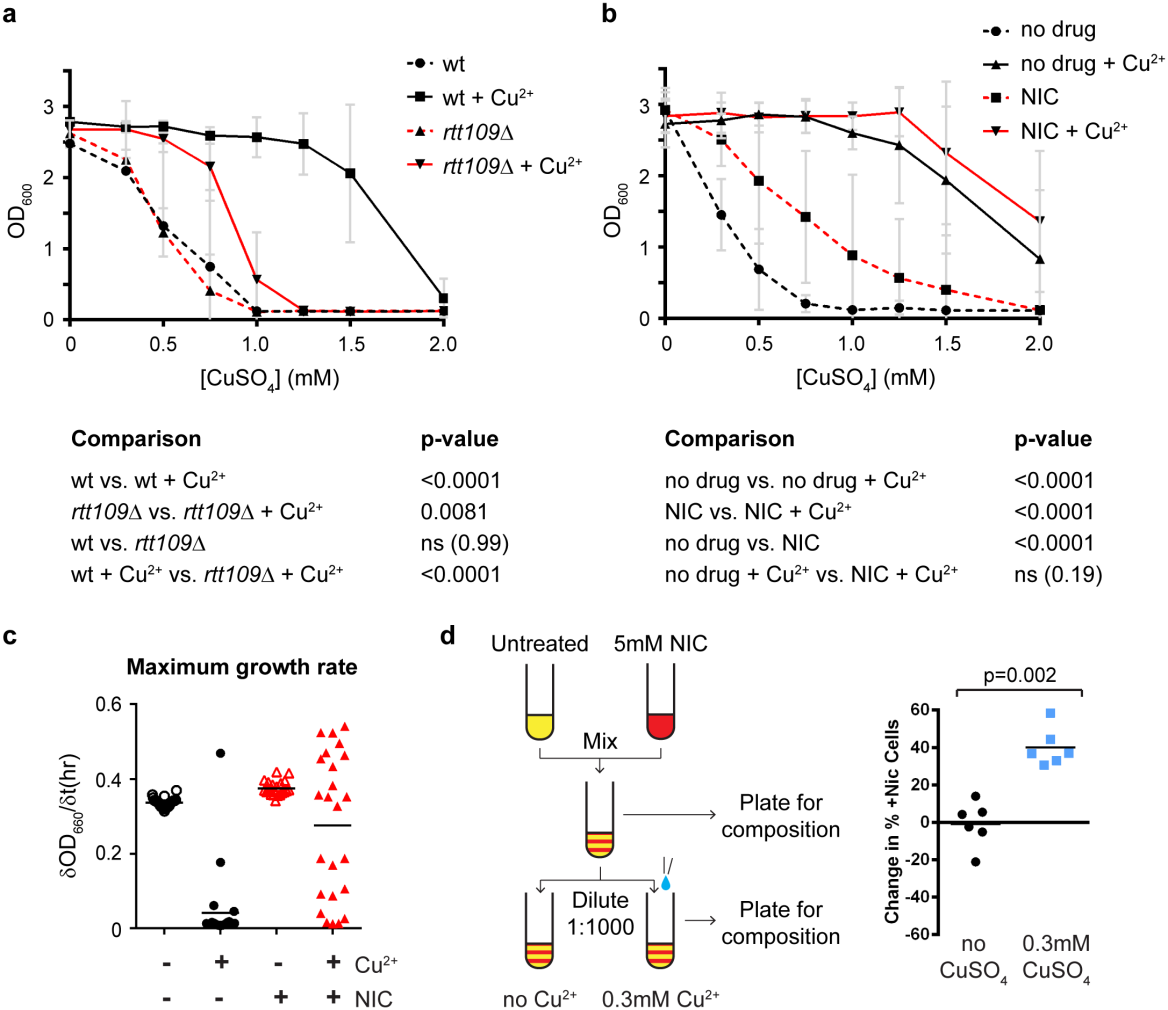
Copper adaptation through stimulated copy number variation (CNV). **a**: Copper resistance of 3x*CUP1* wild-type (wt) and *rtt109*Δ cells grown with or without 0.3 mM CuSO_4_ from Fig 6d. Cells were diluted in media with varying concentrations of CuSO_4_ and grown for 3 days. Average OD_660_ is plotted, error bars represent ±1 SD, and *n* = 6 cultures per condition, each tested at 8 CuSO_4_ concentrations. *p*-values were calculated by 1-way ANOVA of area-under-curve values for each culture. **b**: Copper resistance of 3x*CUP1* cells grown with or without 5 mM nicotinamide (NIC) and with or without 0.3 mM CuSO_4_ from Fig 6c. Analysis as in **a**; *n* = 12. **c**: Maximum growth rate in 0 mM or 0.75 mM CuSO_4_ of 3x*CUP1* cells pretreated with or without 5 mM nicotinamide for 10 generations. dOD_660_/dt represents the OD change per hour. Four samples each grown with or without nicotinamide were each inoculated in 6 cultures for growth curve determination across 72 hours. Data are the maximum of the first derivative of smoothed OD_660_ time-course data (see S7c Fig) for each culture. **d**: Competitive growth assay in 0 or 0.3 mM CuSO_4_. Two populations of 3x*CUP1* cells with different selectable markers were pregrown with or without 5 mM nicotinamide, then mixed and outgrown for 10 generations in direct competition. The graph shows the change in composition of outgrowth cultures across the competition period between inoculation and saturation (10 generations). *p*-value was calculated by paired *t* test, *n* = 6. Raw quantitation data are available in S7 and S8 Data.

To assess this, we initially tested the copper resistance of the 3x*CUP1* wild-type and *rtt109*Δ cells grown with or without 0.3 mM CuSO_4_ shown in Fig 6d. Growth of wild-type cells in copper caused a dramatic rise in copper resistance, with GI_50_ (concentration of CuSO_4_ causing a 50% inhibition of growth) rising >3-fold from 0.5 mM in untreated cells to ~1.7 mM in cells pregrown in 0.3 mM CuSO_4_ (Fig 7a). This increase was clearly attributable to CNV, as cells that grew in 1 mM CuSO_4_ carried large *CUP1* amplifications (S7a Fig). In contrast, *rtt109*Δ cells underwent a far smaller increase, rising <2-fold from 0.5 mM to ~0.8 mM after pregrowth in 0.3 mM CuSO_4_ (Fig 7a), and of the *rtt109*Δ cells that did survive at 1 mM CuSO_4_, albeit with much reduced growth compared to wild-type cells, only a fraction had undergone *CUP1* amplification, suggesting that most had acquired resistance through other, potentially random, mutations (S7a Fig). This shows that acquisition of copper resistance, far from being inevitable, is strongly dependent on an Rtt109-dependent amplification mechanism.

We similarly assessed the effect of nicotinamide on copper resistance using the cells grown with or without CuSO_4_ and with or without nicotinamide shown in Fig 6c. The additive effect of nicotinamide and copper provided a small and not significant increase to the already substantial copper resistance of cells pregrown in 0.3 mM CuSO_4_, but more surprisingly, pregrowth in nicotinamide caused a 2.5-fold increase in GI_50_ for CuSO_4_, from 0.3 mM to 0.75mM (Fig 7b). This is in contrast to the Southern blotting data, which show that the primary effect of nicotinamide treatment is copy number loss (Fig 6b and 6c) and suggest that a substantial amount of amplifications are also generated. In support of this, the *CUP1* copy number of nicotinamide pretreated cells that grew in 0.75 mM CuSO_4_ was substantially amplified (S7b Fig), showing that nicotinamide treatment increases copper resistance by promoting *CUP1* amplification.

These striking effects of H3K56 acetylation on copper resistance suggested that CNV stimulation should provide substantial selective advantages at the population level. As nicotinamide treatment induced constitutive stimulated CNV in the absence of copper, we used this drug to directly test the selective benefit provided by stimulated CNV.

Firstly, we examined strong copper selection based on growth in 0.75 mM CuSO_4_, a concentration fully inhibitory to growth of 3x*CUP1* cells. We pretreated four 3x*CUP1* cultures for 10 generations with or without 5mM nicotinamide, then obtained 6 growth curves for cells from each culture with or without 0.75mM CuSO_4_ in the absence of nicotinamide (S7c Fig). Maximum growth rates were derived from each growth curve to determine whether any cells had adapted sufficiently to allow as rapid growth in the presence of 0.75 mM CuSO_4_ as in the absence of copper (Fig 7c). Nicotinamide pretreatment had no effect on growth rate in the absence of copper but dramatically increased adaptation to copper: only 1 of the 24 cultures grown in the presence of 0.75 mM Cu without nicotinamide pretreatment grew normally, whereas over half (13 of 24) cultures derived from the nicotinamide pretreated samples reached growth rates equivalent to cells growing in the absence of copper. This was not due to rare events in a few of the precultures because the distribution of growth rates obtained in samples from each pretreated culture was similar (S7d Fig). Therefore, although CNV stimulation primarily causes copy number contraction, it also dramatically enhances the ability of a subpopulation of cells to thrive in otherwise toxic concentrations of copper.

Secondly, we asked whether stimulated CNV provides a competitive advantage in low-copper environments in which nonamplified cells are still capable of growth. Under these conditions, the fact that stimulated CNV primarily causes copy number reduction (and therefore further slows growth in copper-containing environments) may put a population of cells using stimulated CNV at a disadvantage relative to a population that does not. To test this, we again made use of the nicotinamide to mimic the effect of stimulated CNV prior to growth in copper-containing media, and we directly competed 1:1 mixtures of untreated and nicotinamide pretreated populations in the same cultures, with or without 0.3 mM CuSO_4_. Treated and untreated populations carried different selectable markers to allow the composition of the mixture to be determined by plating before and after growth (Fig 7d, left). Nicotinamide pretreatment did not alter the competitive fitness of cells in the absence of copper, but the nicotinamide-treated populations efficiently outcompeted the untreated populations in the presence of 0.3 mM CuSO_4_, increasing their population share by 40% on average over 10 generations (Fig 7d right). These experiments clearly show that although stimulated CNV engenders many more contractions than amplifications, it still provides a major selective advantage in both low- and high-copper environments.

Together, our findings demonstrate that bidirectional promoter induction in the *CUP1* genetic context can stimulate CNV to form novel adaptive alleles and that the rate of stimulated CNV is responsive to a controllable histone modification system. Stimulated CNV provides a clear selective advantage, and amplified alleles conferring improved resistance arise in low-copy strains by a mechanism consistent with CNV stimulation.

## Discussion

The assertion that adaptation occurs purely through natural selection of random mutations is deeply embedded in our understanding of evolution. However, we have demonstrated that a controllable mechanism exists in yeast for increasing the mutation rate in response to at least 1 environmental stimulus and that this mechanism shows remarkable allele selectivity. This mechanism has the potential to act widely in eukaryotic genomes, even if restricted to repeated sequences, and may therefore underlie a substantial fraction of observed CNV events.

### A proposed mechanism for stimulated CNV

**Fig 8:**
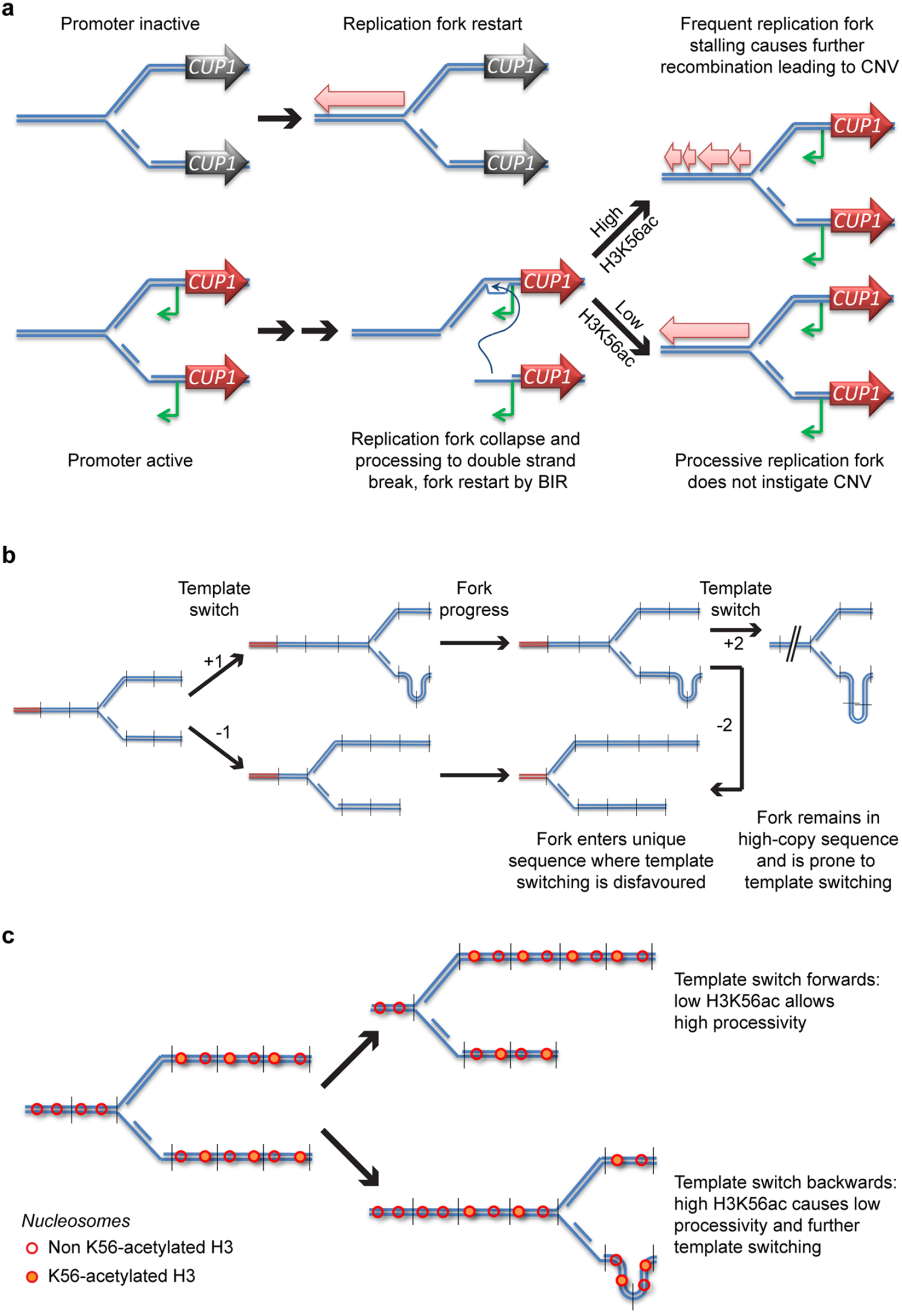
A scheme for stimulated copy number variation (CNV). **a**: Proposed mechanism by which promoter activity and acetylated histone H3 lysine 56 (H3K56ac) contribute to stimulated CNV. DNA strands are shown in blue, the inactive *CUP1* gene is shown in black, and the induced *CUP1* gene is shown in red, with antisense CUT in green. Pink block arrows represent progression of the replication fork. **b**: Proposed mechanism for CNV asymmetry. DNA strands from repeated DNA are shown in blue, unique sequences are shown in red, and vertical lines indicate repeat boundaries. Numbers indicate the change in copy number for a particular template switch. The result of 2 successive template-switching events with an intervening period of replication are shown, resulting in a 3:1 ratio of contractions to amplifications. **c**: Additional asymmetry is generated by H3K56ac: nucleosomes around a replication fork (blue) are shown as red circles, either empty or shaded to represent the H3K56ac state. Template switching forward is seen to move the fork to a region of low H3K56ac, whereas template switching backwards moves the fork to a region of high H3K56ac and therefore low fork stability. BIR, break-induced replication.

We propose a model for stimulated CNV in which local bidirectional promoter activity destabilises stalled replication forks, increasing the frequency of error-prone BIR events. Replication fork stalling occurs widely though nonrandomly in the yeast genome, but stalled forks are normally resolved through error-free mechanisms that protect genome stability (Fig 8a, top left) (reviewed in [81]). However, we suggest that induction of transcription from an adjacent bidirectional promoter increases the likelihood that stalled replication forks collapse (Fig 8a, bottom left); this may occur either through direct interference of the transcription machinery with the stalled fork or indirectly through increased topological stress. Either way, the collapsed fork must then be restarted by a mechanism such as BIR (Fig 8a, bottom middle), forming a replication fork with reduced processivity that is prone to CNV, particularly when H3K56ac is high (Fig 8a, right). This mechanism is closely related to the fork-stalling and template-switching (FoSTeS) process that is suggested to underlie a wide range of CNV events [82], but with contributions from transcription and H3K56ac. Our data implicating H3K56ac and Pol32 in *CUP1* CNV is consistent with restart by BIR, since the replication forks newly formed through BIR are error-prone in the absence of Pol32 or the presence of H3K56ac [70–72]. It is worth noting, however, that other error-prone replication fork restart mechanisms are known, and these may have similar dependencies [83, 84].

This mechanism would be expected to yield both expansions and contractions, and we suggest that 2 additional factors drive the contraction bias we actually observe. Firstly, a fork restarted by HR within a high-copy sequence has a high chance of template switching through strand invasion of a homologous sequence in a different copy, whereas a unique sequence provides only a single homologous template and so template switching is disfavoured. Copy number amplification requires the fork to template switch backwards and re-replicate multiple copies (Fig 8b, upper), whereas contraction requires a template switch forwards that moves the fork closer to the end of the high-copy sequence (Fig 8b, lower). A fork that has switched backwards will therefore spend longer replicating a high-copy sequence than a fork that has switched forwards and would have a higher chance of template switching again, with further potential to generate a contraction (Fig 8b, right). The scheme in Fig 8b shows that 2 successive template-switching events can result in a 3:1 ratio of contractions to amplifications.

This factor would prevail in nicotinamide-treated cells in which H3K56ac is uniformly high, but in normal cells, H3K56ac is primarily on the newly synthesised histones behind the fork and so H3K56ac is also asymmetrical (Fig 8c). This means that a template switch forwards would lead to BIR using a previously unreplicated template with low H3K56ac and would result in a high-processivity replication fork (Fig 8c, upper). In contrast, a template switch backwards would result in the BIR fork using a previously replicated template with high H3K56ac, and this fork would have low processivity and a higher chance of template switching again (Fig 8c, lower). Together, we believe that these 2 factors would yield a major bias towards contraction events but would not prevent amplifications occurring at a lower frequency.

We initially hypothesised that CNV at the *CUP1* locus would be mechanistically equivalent to CNV at the rDNA. Indeed, the requirement for bidirectional promoter induction and the effect of H3K56 HDACs are similar in the 2 systems. However, we see important differences: a lack of dependence on Sir2, which is not surprising as Sir2 acts primarily at heterochromatin; and the suppression of *CUP1* CNV in *rtt109*Δ cells, which conversely undergo massive rDNA amplification [25, 26]. The mechanistic analysis of rDNA amplification in *rtt109*Δ mutants by the Kobayashi group [26] provides an explanation for this discrepancy: rDNA amplification in *rtt109*Δ mutants does not proceed via chromosomal BIR; instead, rolling circle amplification of extrachromosomal rDNA circles (ERCs) forms large arrays of additional rDNA copies that can reintegrate into the chromosomal rDNA locus [26]. *CUP1* circles have been detected but are 3–4 orders of magnitude rarer than ERCs [85], and we suspect that rolling circle amplification of these happens in too few cells to make a detectable contribution.

### Adaptive potential of stimulated CNV

Stimulated CNV controls the occurrence of a subset of mutations that allow adaptation to challenging environments. It is commonly assumed that adaptive mutations occur at random, and they are largely inevitable as they occur through multiple poorly defined mechanisms. Under this assumption, loss of many genome stability factors would increase the rate of adaptation, but adaptation cannot be suppressed. In contrast, we show that the adaptation of yeast to environmental copper by amplification of *CUP1* is largely dependent on a defined pathway that requires Rtt109, despite the general role of this protein in maintaining genome stability. Therefore, adaptation occurring through apparently random mutation may in fact be stimulated by a specific cellular mechanism.

How widespread is stimulated CNV? The *CUP1* and *SFA1* model systems we have analysed are multicopy, but although CNV will be most efficient where multiple homologous sequences surround the RFS site, this mechanism should not be restricted to multicopy loci. Recombination events triggered by error-prone replication forks could easily utilise distal homologous sequences or even microhomology as templates, inducing de novo CNVs and chromosomal translocations with limited homology at the breakpoints. Breakpoints in de novo CNVs would therefore be poorly defined because they initiate nearby but not at the RFS site and utilise unpredictable homologous sequences. Interestingly, *CUP1* shows exactly this behaviour in different *S*. *cerevisiae* isolates, as the multicopy *CUP1* repeat has emerged many times with different breakpoints [58].

Replication fork stalling is by no means restricted to budding yeast, and bidirectional promoters are the norm in organisms, including mammals [86, 87]. Therefore, the basic machinery required for stimulated CNV is likely to be conserved. Furthermore, the histone deacetylases that regulate CNV outcome are conserved in mammals and appear to have similar functions in modulating DNA repair [88, 89]. Stimulated CNV in somatic cells of metazoans is rarely likely to be a useful organismal outcome and cannot aid heritable adaptation. However, because stimulated CNV emerges from conserved features of the replication and transcription systems, it seems likely that it would be active in mammalian cells, providing a mechanism that could be readily exploited, for example, by tumour cells. The mechanism that we have proposed is also very consistent with recent reports of nonrandom double-strand breaks formed by neurons in genes important for neuronal function [90, 91]. As such the stimulated CNV pathway provides a new set of targets by which pharmaceuticals may prevent the emergence of undesirable properties such as drug resistance in tumours or modulate natural genetic changes in particular cell types. Indeed, our observation that adaptation of yeast to copper can be effectively suppressed by removal of Rtt109, a protein for which chemical inhibitors have been described [92, 93], provides good evidence that the emergence of resistance is pharmacologically accessible.

Evidence for adaptation through genome-wide nonrandom mutation is substantial, particularly in bacteria [18], but the ability of stimulated CNV to direct mutations to relevant loci must be reconciled with forceful arguments against previously proposed ‘directed mutation’ systems [18, 30, 94, 95]. The primary issue is that any general mechanism that directs mutations to a particular site must ‘know’ in advance the fitness outcome of a particular genetic change, which is not possible except at singular, highly specialised loci such as the rDNA. However, such arguments ignore the wealth of information regarding the function of particular loci in particular environments that is encapsulated in existing gene regulatory systems. In effect, a signalling pathway that strongly induces a gene in response to a particular environmental stimulus marks that gene as being important in that environment relative to a gene that is not expressed. CNV of such a gene is more likely to yield a useful, adaptive result than CNV of a random gene. Of course, it is also more likely to be damaging, and we see exactly this at *CUP1*: most of the CNV events we observed were contractions that reduce copper resistance (Fig 3b), but, relative to random mutation, the chance of finding an adaptive CNV remains substantial. Stimulated CNV is therefore a high-risk strategy that does not entail foreknowledge of the fitness outcome of genetic change at a particular locus, only the relative importance of that locus in the current environment.

However, many highly expressed genes are not environment specific, and such housekeeping genes are likely to be poor candidates for improving adaptation. Simply focusing CNV events at highly expressed genes would likely entail an unacceptable number of deleterious CNV events involving housekeeping genes. This problem is avoided by restricting RFS sites to the promoters of inducible genes. We suggest that the distribution of RFS sites has arisen through natural selection acting on randomly located RFS sites, as any RFS site that always engenders detrimental CNVs would have been rapidly lost.

Stimulated CNV is therefore an imperfect but useful cellular mechanism that increases the rate at which adaptive CNV events occur, particularly in suboptimal environments. Importantly, inducible gene expression systems and the placement of RFS sites are products of natural selection acting on random mutations, but these combine to yield a system that accelerates adaptation beyond what is achievable through random mutation.

## Materials and methods

### Yeast strains and media

Yeast strains used in this work are listed in S2 Table. Plasmids are listed in S3 Table, including construction details, and were verified by restriction digest and/or sequencing. Deletion strains were created by standard methods; oligonucleotides are listed in S4 Table. Deletion strains were verified by PCR. To create the *P*_*GAL1*_*-HA* strain, *ADE2* was replaced with *MET25* in the *S*. *cerevisiae* strain BY4741 (EuroSCARF), and the resulting strain was transformed with pJH252 (1 *CUP1* repeat); then, the entire repeated region at the chromosomal *CUP1* locus [ChrVIII: 212265..216250] was replaced with a *LEU2* marker. Plasmid pJH280, containing 3 copies of the *P*_*GAL1*_*-HA* construct and an *ADE2* marker, was digested with *Sac*I and transformed to replace the *LEU2* cassette, which fortuitously amplified on transformation to yield a 17-copy repeat tract (based on PFGE Southern blot migration). The 17x*P*_*GAL1*_*-3HA* construct was introduced into the MEP background by mating and sporulation and was remated to a MEP wild-type haploid to form the MEP 17x*P*_*GAL1*_*-3HA* heterozygote strain.

For construction of the 3x*CUP1* strain and its derivatives, the entire *CUP1* locus [ChrVIII: 212265..216250] was deleted in YRH12, an *ade2*Δ BY4742-derivative with a single-copy *CUP1* plasmid, to form YRH15. pRH9, which contains 3 complete *CUP1* copies [ChrVIII: 214256..216239] with an *ADE2* marker and *CUP1* flanking sequences, was digested with *Sac*I and transformed in YRH15, followed by FOA selection to yield YRH23.

Construction of the 3x*SFA1* strain and its derivatives used the same strategy as for 3x*CUP1*, except that *SFA1* was additionally deleted in YRH15 and then transformed with *Sac*I-digested pJH312, followed by FOA selection to yield YRH89.

Cells for Fig 2c and 2d were grown in YPD, and other experiments were performed in yeast nitrogen base media supplemented with CSM amino acids and 2% glucose or galactose; all cells were grown at 30°C. YPD contains trace Cu^2+^, and yeast nitrogen base media contains 250 nM CuSO_4_. All media components were purchased from Formedium. Nicotinamide (Sigma I17451) was added to media at 5 mM. For Fig 6b–6d, cells were grown in SC with or without 0.3 mM CuSO_4_ in 4-ml cultures, diluted 1:1,000 from saturated precultures. For Fig 6e and 6f, cells were grown to log phase in SC then diluted 1:8,000 into SC with or without 0.9–1 mM (batch-dependent) formaldehyde that was freshly diluted from 16% or 37% stock solution. Fig 2a represents a meta-analysis of published data; see the Bioinformatic analysis section below for accession numbers and associated culture details.

For cell-tracking analyses using the MEP system, cells were inoculated in SD from a plate for 6–8 hours, diluted and grown overnight to OD 0.2–0.5. Cultures were diluted to 2x10^4^ cells/ml, 1 μM β-estradiol (Sigma E2758) was added, and cells were grown for 2 hours prior to plating parental culture and splitting the cells for copper or galactose treatment. After 24 hours, 50 μl of each culture was plated on SD agar, and cells were grown for 2 days at 30°C. Individual colonies were then inoculated in 200 μl of SD in a 96-well plate and grown to saturation.

### Copper sensitivity and growth curve analysis

For the MEP strain, 2.5 μl saturated culture was diluted to 200 μl SC in each well of a 96-well flat-bottomed cell culture plate, with concentrations of CuSO_4_ up to 3 mM, along with 0.5 mM ascorbic acid. Ascorbate increases the cellular uptake of copper [96], increasing the effective toxicity of copper to allow the measurement of small changes in resistance in cells with high *CUP1* copy number. This is helpful since CuSO_4_ tends to precipitate out of media during culture at concentrations >2 mM. Plates were covered with a gas-permeable membrane and grown at 30°C for 3 days in the dark. Cells were resuspended by pipetting, and OD_660_ was measured using a BD FLUOstar Omega plate reader. Area-under-curve measurements were calculated for each sample and compared by 1-way ANOVA. For the 3x*CUP1* strain, the assay was performed as above but with lower concentrations of CuSO_4_ (see Fig 7a and 7b), under normal light and without ascorbic acid.

For growth curves, saturated precultures were diluted 1:1,000 into 200 μl SC per well with or without CuSO_4_ at the required concentration. Plates were sealed as above and grown at 30°C with shaking in a BD FLUOstar Omega plate reader; OD_660_ measurements were taken every 15 minutes. Curves were smoothed by averaging across 9 time points, and derivatives were calculated using GraphPad Prism.

### Competitive growth assay

Six cultures each of 3x*CUP1* wild-type and *trp1*Δ::NatMX6 were grown for 10 generations, 3 untreated and 3 with 5 mM nicotinamide. Cultures were then mixed 1:1 pairwise to give 6 competition cultures, each containing an untreated and a nicotinamide pretreated population of the opposite genotype. The composition of the mixture was determined by plating on–Trp and +Nat plates, and each mixture was inoculated 1:1,000 in cultures containing 0 or 0.3 mM CuSO_4_ and outgrown to saturation over 10 generations. Mixture composition of each outgrowth culture was determined by plating. To ensure that the *trp1*Δ::NatMX6 marker did not affect the result, we performed equal numbers of assays with this strain as the nicotinamide-treated or untreated population.

### DNA extraction and Southern blotting

Cells from a 2 ml saturated culture were washed with 50 mM EDTA then spheroplasted with 250 μl 0.34U/ml lyticase (Sigma L4025) in 1.2 M sorbitol, 50 mM EDTA, and 10 mM DTT at 37°C for 45 minutes. After centrifuging at 1,000g, cells were gently resuspended in 400 μl of 0.3% SDS, 50 mM EDTA, and 100μg/ml RNase A (Sigma R4875) and incubated at 37°C for 30 minutes. 4 μl of 20 mg/ml proteinase K (Roche 3115801) was added, and samples were mixed by inversion and heated to 65°C for 30 minutes. 160 μl 5M KOAc was added after cooling to room temperature, and samples were mixed by inversion and then chilled on ice for 30 minutes. After 10 minutes of centrifuging at 20,000g, the supernatant was poured into a new tube containing 500 μl phenol:chloroform (pH 8) and samples were mixed on a wheel for 30 minutes. Samples were centrifuged for 10 minutes at 10,000g, and the upper phase was extracted using cut tips and precipitated with 400 μl isopropanol. Pellets were washed with 70% ethanol, air-dried, and left overnight at 4°C to dissolve in 20 μl TE. After gentle mixing, 10 μl of each sample was digested with 20 U *Eco*RI-HF (NEB) or *Eco*RV-HF (NEB) for 3 hours, phenol:chloroform extracted, ethanol precipitated, and separated on 25-cm 0.8% or 1% 1xTBE gels overnight at 120 V for *CUP1* analysis or on 1% 0.5xTBE gels in a Bio-Rad CHEF DR-III system at 6 V/cm, 15°C, 0.5–1.5 second switch, and 120° included angle for 16 or 20 hours in 0.5xTBE for *SFA1* analysis. Gels were washed in 0.25 N HCl for 15 minutes, 0.5 N NaOH for 45 minutes and twice in 1.5M NaCl, 0.5M Tris (pH 7.5) for 20 minutes before being transferred to HyBond N+ membrane in 6x SSC. Membranes were probed using random primed probes (S4 Table) in UltraHyb (Life Technologies) at 42°C and washed twice with 0.1 x SSC, 0.1% SDS at 42°C. Bands were quantified using ImageQuant (GE) and data analysed using the GraphPad Prism v6.05 to perform 1-way ANOVA analyses comparing the means of all samples (unless otherwise noted) with Tukey correction for multiple comparisons.

### Rapid DNA extraction and PFGE for cell fate tracking analysis

Colonies were analysed in pools of 4. Cells obtained from 50 μl from each of the 4 saturated cultures were resuspended in 50 μl 50 mM EDTA containing 17 U lyticase (Sigma L2524) and incubated at 37°C for 45 minutes. 1.6 μl 10% SDS and 1 μl 20 mg/ml proteinase K were added and samples were incubated at 65°C for 30 minutes. After addition of 32 μl 5 M KOAc and 30 minutes on ice, samples were centrifuged for 10 minutes at 20,000g at room temperature, and the supernatant was decanted to a new tube containing 100 μl isopropanol and 1 μl glycogen. Samples were centrifuged for 15 minutes at 20,000g at 4°C, and the pellet was washed with 70% ethanol before overnight elution in 20 μl 1x NEB CutSmart buffer with 20 U *Eco*RI-HF (NEB) at 37°C. DNA was quantified using PicoGreen (Thermo Fisher Scientific) and separated on PFGE gels using a Bio-Rad CHEF DR-III (1% 0.5xTBE gel, 6 V/cm, 15°C, 0.5–1.5 second switch, 120° included angle for 20 hours in 0.5xTBE), then blotted and probed as above. Copy numbers of individual alleles were plotted using GraphPad Prism v6.05.

To calculate *p*-values, we first estimated the background CNV or amplification mutation rate in the population based on the number of CNV or amplification events observed by PFGE for the unstimulated condition, including the viability of this population after 24 hours of aging. Using this estimate, we then calculated the number of CNV or amplification events in the stimulated condition that would be expected to arise through unstimulated CNV given the viability of this population. We then compared the number of events observed by PFGE to the expected number of CNV or amplification events for the stimulated condition using a goodness of fit χ^2^ test with 1 degree of freedom. This provides a *p*-value based on the null hypothesis that all observed CNV or amplification events arose through random mutation. This estimate includes the conservative assumption that any cells that lost viability during the experiment did not undergo CNV.

### RNA extraction and northern analysis

Total RNA was extracted using a mirVANA kit (Thermo Fisher Scientific) according to manufacturer’s instructions (Fig 2) or using GTC-phenol as described [7] (Figs 4, 5 and 6), and analysed as previously described [7] using probes listed in S4 Table. RNA probes were hybridised at 65°C, DNA probes at 42°C. Indexed mRNAseq libraries were constructed from 500 ng total RNA using the NEBNext Ultra Directional RNA Library Prep Kit (NEB), with poly(A) selection using the NEBNext Poly(A) mRNA Magnetic Isolation Module (NEB), and sequenced on an Illumina MiSeq.

### Chromatin immunoprecipitation

0.5x10^9^ cells grown in YPD, with or without 4 hours of 1 mM CuSO_4_ treatment, were fixed for 15 minutes in 1% formaldehyde and quenched with 150 mM glycine. Cells were washed 2 times with cold PBS, then resuspended in 600 μl lysis buffer (50 mM HEPES [pH 7.5], 140 mM NaCl, 1 mM EDTA, 1% Triton X-100, 0.1% Na-deoxycholate, 0.1% SDS, 1x Roche Complete Protease Inhibitors), broken with 500 μl 0.5mm zirconium beads (BioSpec) in an MP Biomedical Fast Prep (6 cycles, 30 seconds each), then the lysate was separated from the beads and diluted to 1 ml final volume in lysis buffer. Samples were sonicated 19 times, 30 seconds each in a Diagenode Bioruptor on High and cleared by centrifugation at 20,000g at 4°C for 5 minutes. 1 μl anti-γH2A (Millipore 07-745-I) was added to 100 μl lysate and incubated overnight at 4°C before addition of 15 μl Gammabind beads (GE) in 25 μl lysis buffer (preblocked with 1% BSA) and incubation for 2 hours at 4°C. Beads were washed 5 minutes each with lysis buffer, 0.5 M salt lysis buffer, wash buffer (10 mM Tris [pH 8.0], 0.25 M LiCl, 0.5% NP-40, 0.5% Na-deoxycholate, 1 mM EDTA), and TE, then DNA was eluted overnight at 65°C in 200 μl 50 mM Tris [pH 8.0], 10 mM EDTA, 1% SDS. DNA was purified by phenol:chloroform extraction and then ethanol precipitated and eluted in 50 μl TE. Sequencing libraries were prepared from 5 ng of immunoprecipitated material using a NEBNext DNA Ultra kit (NEB) and sequenced on an Illumina HiSeq.

### Bioinformatic analysis

γH2A ChIP: Reads were mapped to the *S*. *cerevisiae* reference genome R64-2-1 using Bowtie 2 v2.2.5 (default parameters). Peaks were called using MACS2 v2.1.0 (-g 12e6—nomodel—extsize 250—keep-dup all), artifactual peaks containing a single mismapped read were manually removed, and only peaks with a 2-fold or greater enrichment were considered. Coding sequences (CDS) were categorised as upstream-RFS if a γH2A peak was present in 1 kb upstream of the annotated start site using the R script in S1 Text.

RNAseq: Read data (including accessions GSE61783 [97], GSE74642, GSE70835, GSE54831 [98], GSE54825 [99], GSE43002 [100], and GSE41834 [101] deposited at GEO) were mapped to genome R64-2-1 using HISAT2 v2.0.5 (—sp 1000,1000). Log_2_ read counts were performed for each annotated CDS using Seqmonk v0.34.1 and normalised for CDS length, then the whole data set was normalised for a median expression of 9 (an arbitrary value that maintained most expression data as positive and required minimal scaling for most data sets). Genes were categorised as γ-H2A or non-γH2A using the R script in S1 Text. Cumulative frequency distributions for the data sets were calculated using GraphPad Prism v6.05. Frequency distributions were compared by nested ANOVA, and assumptions for using parametric tests were checked prior to run the analyses. Values for skewness, kurtosis, and variance were consistent with normality and homoscedasticity.

### Image processing and data availability

Sequencing data has been deposited at GEO (GSE86283). Source data for all graphs is provided in S1 Data (cumulative frequency gene expression data), S2 Data (RNAseq data for cells grown with or without Cu), S3 Data (Southern blot image quantification), S4 Data (northern blot image quantification), S5 Data (γH2A ChIPseq data for chromosomes containing *CUP1* and *SFA1* loci), S6 Data (mother enrichment allele copy numbers and adaptation assay), S7 Data (3x*CUP1* adaptation assays and competition assay), and S8 Data (Raw growth curve data).

Gel images were processed with ImageJ 1.50i—processing involved rotating and cropping, denoising if required (Despeckle filter), and altering window-level settings to improve contrast of relevant bands. Full images of membranes presented in the manuscript are provided in S9 Data—these have been cropped to the borders of the membrane and have undergone minimal window-level adjustments if required to make the bands shown in the presentation figure visible.

## Supporting information

S1 Fig Cumulative frequency distributions showing expression of upstream-RFS genes. **a**: Data from matched set of cells grown in YPD and YPGlycerol, GSE74642, showing cumulative frequency distributions for the expression of genes either with (γH2A) or without (non-γH2A) an upstream stalled replication fork site. The YPGlycerol data set overlaps with some YPD data in Fig 2a but is clearly separable from the matched YPD control. **b**: Data sets as in **a** for cells subjected to oxidative (0.4 mM H_2_O_2_ for 30 minutes) or osmotic stress (0.4 M KCl for 10 minutes). Data were reanalysed from GSE42983 [100] and GSE61783 [97] using the R script provided in S1 Text; raw quantitation data are available in S1 Data.
(TIF)

S2 Fig RNAseq analysis of copper-treated cells. Wild-type cells were grown in YPD with or without 1 mM CuSO_4_ for 4 hours, analysed by poly(A)+ RNAseq, and read counts mapping to each annotated coding sequence (CDS) were calculated and normalised for feature length. **a**: Cumulative frequency distribution showing expression of genes with an upstream γH2A peak relative to control genes under each condition. **b**: Scatterplot of RNA levels, with γH2A genes highlighted in red. CDS for γH2A genes that are substantially induced or repressed by copper are annotated: the blue circle shows *CUP1* locus genes, the green circle shows the single other verified coding sequence induced by copper (*HSP12*), and orange circles are CDS representing the multicopy, subtelomeric helicase ORFs. Raw quantitation data are available in S1 and S2 Data.
(TIF)

S3 Fig Supplement to role of H3K56 acetylation in stimulated CNV. **a**: Southern analysis of *CUP1* copy number in wild-type, *sir2*Δ, and *hst3*Δ *hst4*Δ cells. **b**: Northern analysis of *CUP1* ORF and *CUP1* CUT RNA in log-phase wild-type, *mrc1*Δ, and *pol32*Δ cells. *p*-values are nonsignificant by 1-way ANOVA, *n* = 3. Raw quantitation data are available in S4 Data.
(TIF)

S4 Fig *GAL1* promoter does not respond to nicotinamide. Northern analysis of *HA* ORF and *CUP1* CUT RNA in log-phase *P*_*GAL1*_*-HA* cells grown on glucose or galactose with or without 5 mM nicotinamide. *p*-values were calculated by 1-way ANOVA, *n* = 4. Raw quantitation data are available in S4 Data.
(TIF)

S5 Fig Nicotinamide stimulation of CNV to low copy number. Reanalysis of Southern data from Fig 5b, quantifying *CUP1* alleles with 1–3 copies compared to the total of all alleles. This shows that nicotinamide stimulation is particularly potent in the production of small alleles that presumably arise from multiple CNV events. *p*-values were calculated from pairwise comparisons of negative and positive NIC samples for each GLU or GAL concentration, derived from a 1-way ANOVA of the whole data set. Raw quantitation data are available in S3 Data.
(TIF)

S6 Fig Characteristics of 3x*CUP1* and 3x*SFA1* systems. **a**: Growth curves of 3x*CUP1* cells growing with or without 0.3 mM CuSO_4_. Note that the growth retardation caused by 0.3 mM CuSO_4_ is stronger in the 200 μl 96-well plate cultures used for growth curve analysis than in the 4 ml batch cultures used for Southern blot samples; although cells also grow slowly in 0.3 mM CuSO_4_ under these conditions, almost all cultures reach saturation by 72 hours. **b**: Southern analysis of *CUP1* copy number in 3x*CUP1* cells grown for 10 generations with or without 0.3 mM CuSO_4_. Quantification shows the percentage of amplified alleles; *n* = 6, *p*-value calculated by *t* test. **c**: γH2A signal in the region surrounding *SFA1*; analysis performed as in Fig 2d. **d**: Induction of *SFA1* and upstream antisense transcripts after a 4-hour exposure to 1 mM formaldehyde, assayed by northern blot. 18S rRNA is shown as a loading control. Quantification shows the levels of the indicated RNA species in arbitrary units; *p*-values were calculated by *t* test, *n* = 4. Locations of probes within the *SFA1* repeat are shown in **e**. **e**: Schematic of the wild-type *SFA1* region in the 3x*SFA1* construct, along with the modified *P*_*GAL1*_*-GFP-SFA1* construct. All *SFA1* copies carry this construct in the *P*_*GAL1*_*-GFP-SFA1* strain. Transcriptional start sites indicated by black arrows are approximate. Raw quantitation data are available in S3, S4, S5 and S8 Data.
(TIF)

S7 Fig Supplement to copper adaptation through stimulated CNV. **a**: *CUP1* copy number distribution of 3x*CUP1* wild-type and *rtt109*Δ cells after growth for 10 generations with with or without 0.3 mM CuSO_4_, followed by growth with or without 1 mM CuSO_4_ under adaptation curve conditions, then outgrown without drug. **b**: *CUP1* copy number distribution of 3x*CUP1* cells after growth for 10 generations with 5 mM nicotinamide, followed by growth with or without 0.75 mM CuSO_4_ under adaptation curve conditions, then outgrown without drug. **c**: Individual growth curves of 3x*CUP1* cells preexposed to 5 mM nicotinamide before growth with or without 0.75 mM CuSO_4_. **d**: Maximum growth rate data for +NIC +CuSO_4_ samples from Fig 7d, separated to show distributions of data points derived from the 4 different precultures. Raw quantitation data are available in S8 Data.
(TIF)

S1 Table List of *S*. *cerevisiae* genes with upstream γH2A peaks. Peaks were identified by MACS analysis of γH2A data on unstressed cells growing at log phase in YPD (see Materials and methods). Peaks were matched to annotated coding sequences (CDS) using an R script (provided as S1 Text).
(XLSX)

S2 Table *S*. *cerevisiae* yeast strains used in this study. (DOCX)

S3 Table Newly derived plasmids used in this study. See S4 Table for oligonucleotides used to amplify cloning fragments.
(DOCX)

S4 Table Oligonucleotide pairs used in this study. (DOCX)

S1 Text R script for meta-analysis of gene expression data sets. (R)

S1 Data Cumulative frequency gene-expression data. (XLSX)

S2 Data Gene-expression values derived from RNAseq. (XLSX)

S3 Data Southern blot quantification. (XLSX)

S4 Data Northern blot quantification. (XLSX)

S5 Data γH2A ChIPseq data for chromosomes containing *CUP1* and *SFA1* loci. (XLSX)

S6 Data Mother enrichment allele copy numbers and adaptation assay. (XLSX)

S7 Data 3x*CUP1* adaptation assays and competition assay. (XLSX)

S8 Data Raw data for growth curves. (XLSX)

S9 Data PDF file containing uncropped images of Southern and northern blots. (PDF)

## Abbreviations

γH2A: S139-phosphorylated histone H2A
ARS: autonomously replicating sequence
AU: arbitrary unit
BIR: break-induced replication
ChIP: chromatin immunoprecipitation
ChIPseq: ChIP sequencing
CNV: copy number variation
CUT: cryptic unstable transcript
ERC: extrachromosomal rDNA circle
FA: formaldehyde
GAL: galactose
GLU: glucose
H3K56ac: acetylated histone H3 lysine 56
HDAC: histone deacetylase
HR: homologous recombination
MEP: mother enrichment program
NAHR: nonallelic homologous recombination
ncRNA: noncoding RNA
ns: not significant
rDNA: ribosomal DNA
RFB: replication fork barrier
RFS: replication fork stalling
RNAseq: RNA sequencing
wt: wild type
