# Supplementary figures and images for: Environmental change drives accelerated adaptation through stimulated copy number variation
Source: PLoS Biol. 2017 Jun 27;15(6):e2001333. doi: 10.1371/journal.pbio.2001333 (PMC5486974; doi:10.1371/journal.pbio.2001333)

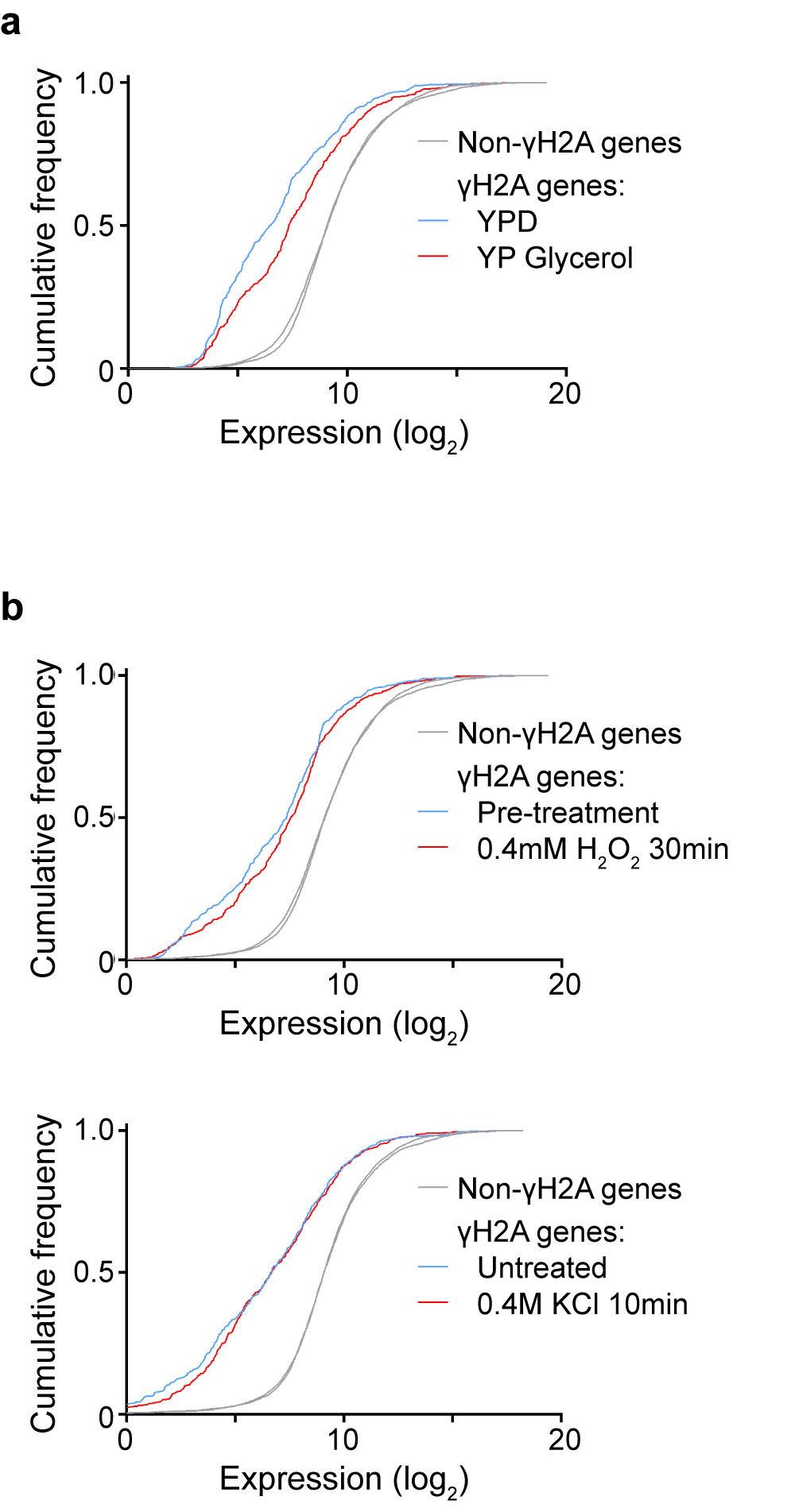

Supplement: S1 Fig — a: Data from matched set of cells grown in YPD and YPGlycerol, GSE74642, showing cumulative frequency distributions for the expression of genes either with (γH2A) or without (non-γH2A) an upstream stalled replication fork site. The YPGlycerol data set overlaps with some YPD data in Fig 2a but is clearly separable from the matched YPD control. b: Data sets as in a for cells subjected to oxidative (0.4 mM H2O2 for 30 minutes) or osmotic stress (0.4 M KCl for 10 minutes). Data were reanalysed from GSE42983 [100] and GSE61783 [97] using the R script provided in S1 Text; raw quantitation data are available in S1 Data. (TIF) [file pbio.2001333.s001.tif]

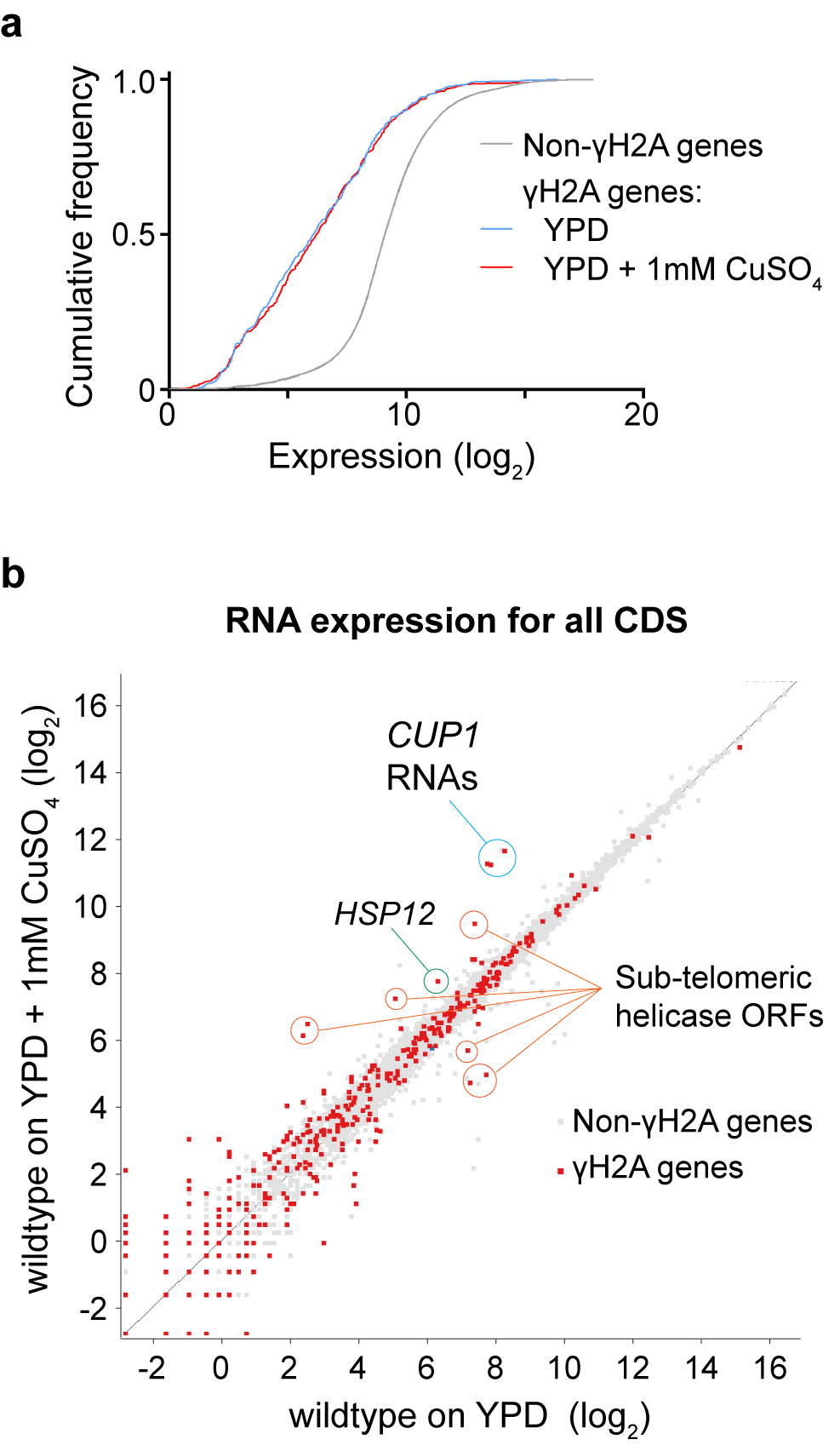

Supplement: S2 Fig — Wild-type cells were grown in YPD with or without 1 mM CuSO4 for 4 hours, analysed by poly(A)+ RNAseq, and read counts mapping to each annotated coding sequence (CDS) were calculated and normalised for feature length. a: Cumulative frequency distribution showing expression of genes with an upstream γH2A peak relative to control genes under each condition. b: Scatterplot of RNA levels, with γH2A genes highlighted in red. CDS for γH2A genes that are substantially induced or repressed by copper are annotated: the blue circle shows CUP1 locus genes, the green circle shows the single other verified coding sequence induced by copper (HSP12), and orange circles are CDS representing the multicopy, subtelomeric helicase ORFs. Raw quantitation data are available in S1 and S2 Data. (TIF) [file pbio.2001333.s002.tif]

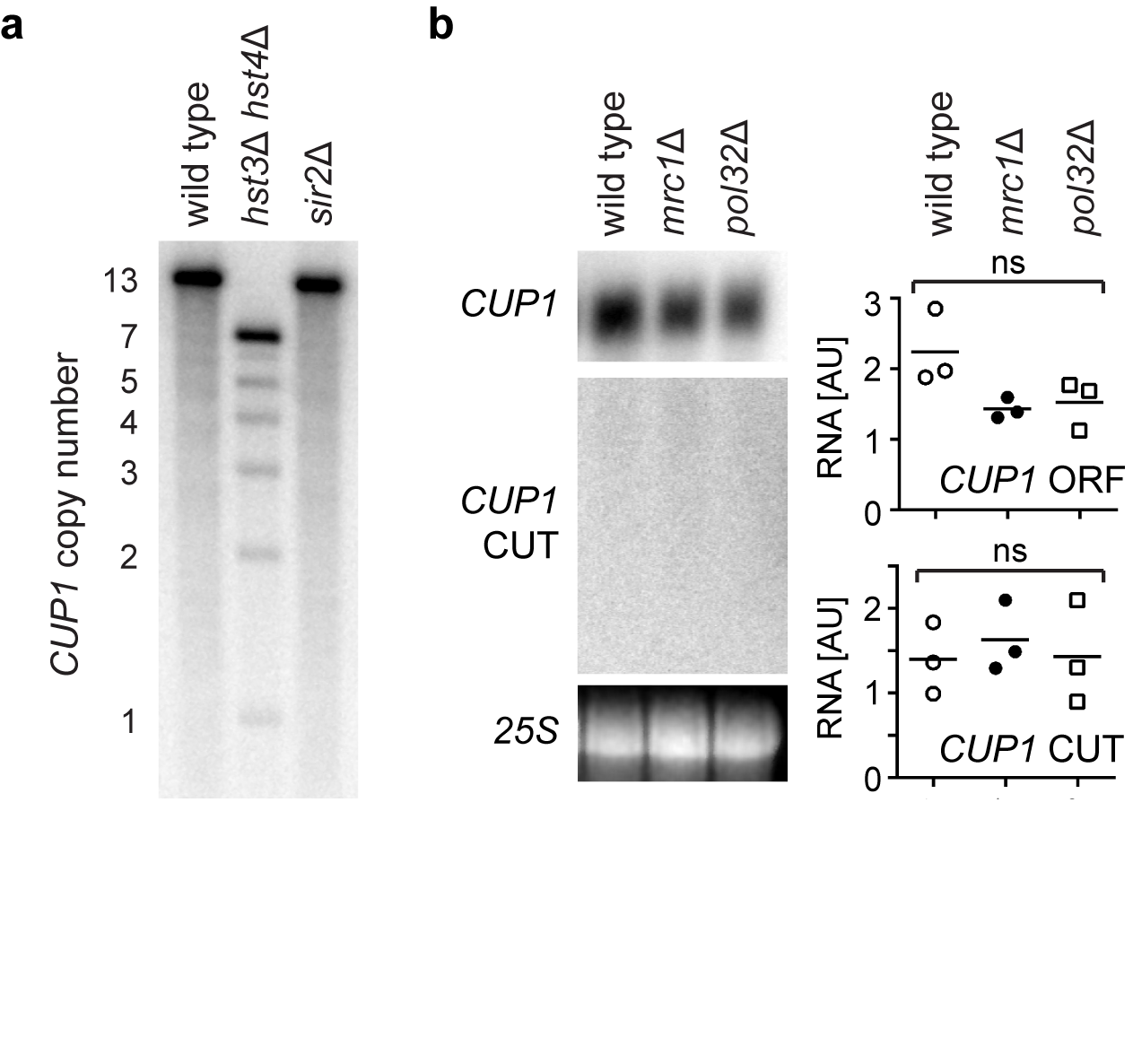

Supplement: S3 Fig — a: Southern analysis of CUP1 copy number in wild-type, sir2Δ, and hst3Δ hst4Δ cells. b: Northern analysis of CUP1 ORF and CUP1 CUT RNA in log-phase wild-type, mrc1Δ, and pol32Δ cells. p-values are nonsignificant by 1-way ANOVA, n = 3. Raw quantitation data are available in S4 Data. (TIF) [file pbio.2001333.s003.tif]

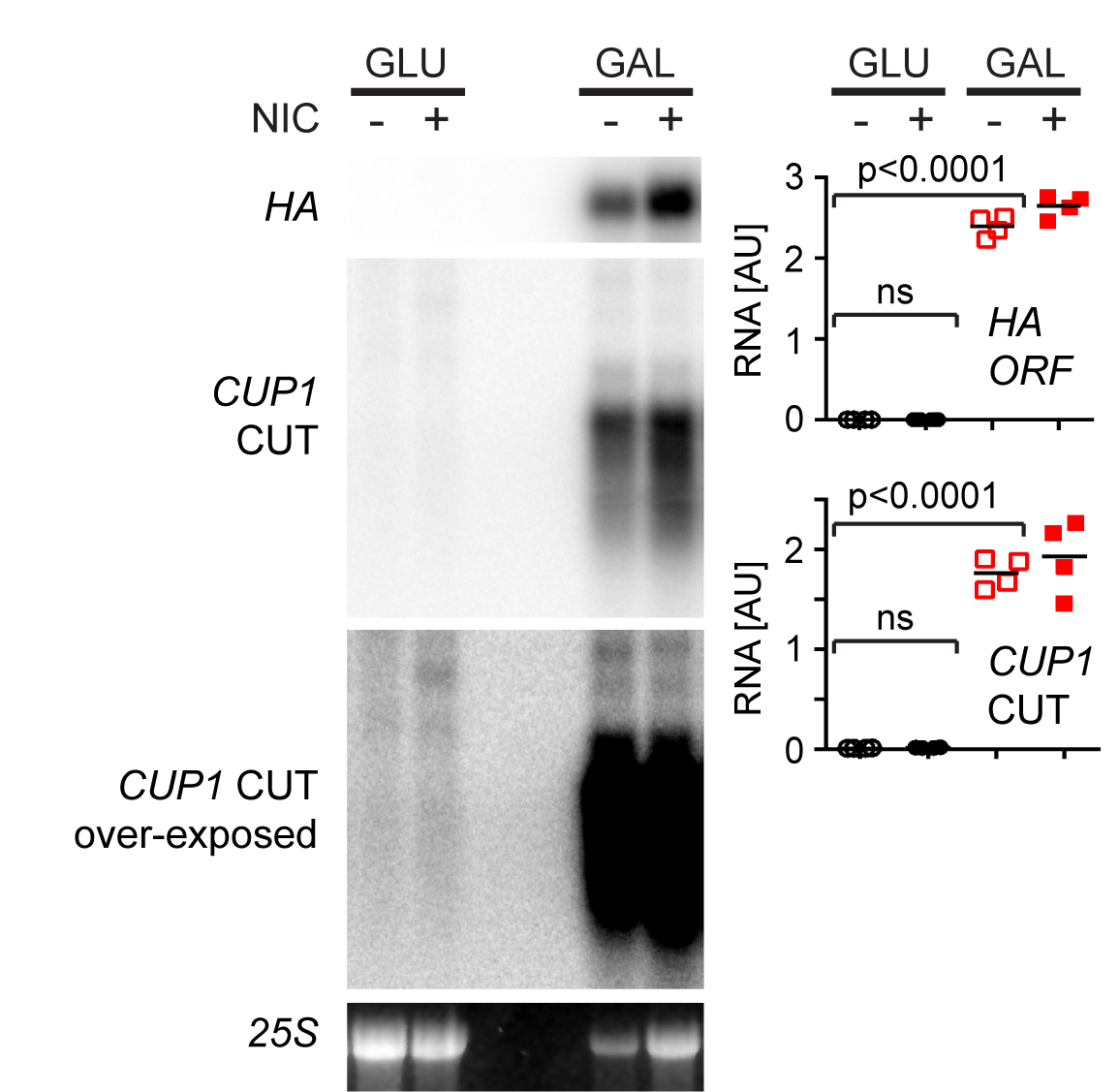

Supplement: S4 Fig — Northern analysis of HA ORF and CUP1 CUT RNA in log-phase PGAL1-HA cells grown on glucose or galactose with or without 5 mM nicotinamide. p-values were calculated by 1-way ANOVA, n = 4. Raw quantitation data are available in S4 Data. (TIF) [file pbio.2001333.s004.tif]

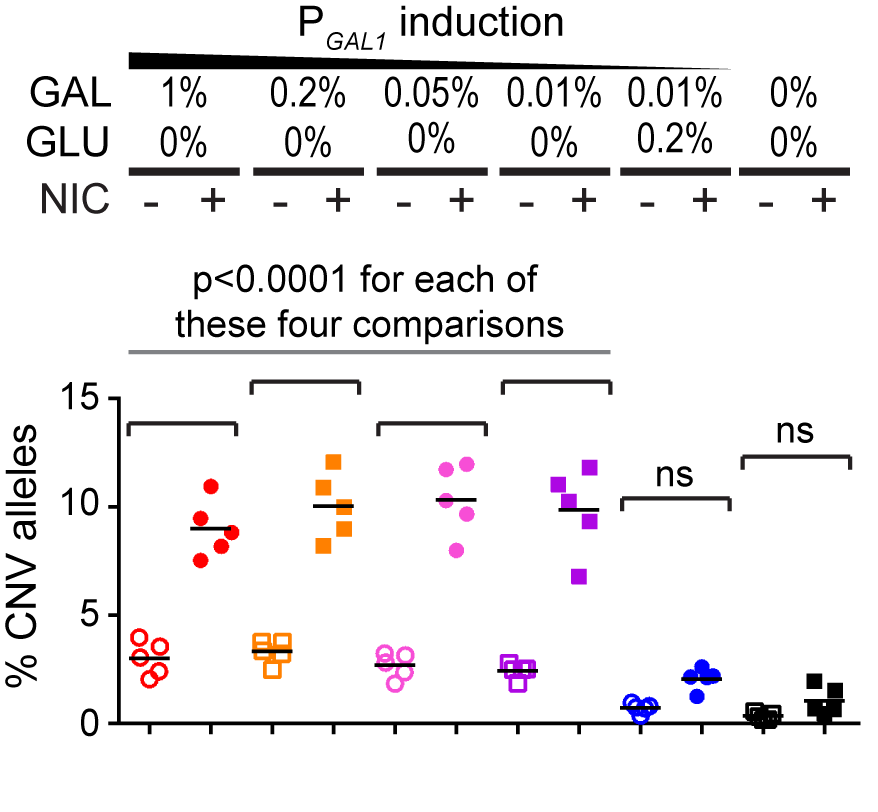

Supplement: S5 Fig — Reanalysis of Southern data from Fig 5b, quantifying CUP1 alleles with 1–3 copies compared to the total of all alleles. This shows that nicotinamide stimulation is particularly potent in the production of small alleles that presumably arise from multiple CNV events. p-values were calculated from pairwise comparisons of negative and positive NIC samples for each GLU or GAL concentration, derived from a 1-way ANOVA of the whole data set. Raw quantitation data are available in S3 Data. (TIF) [file pbio.2001333.s005.tif]

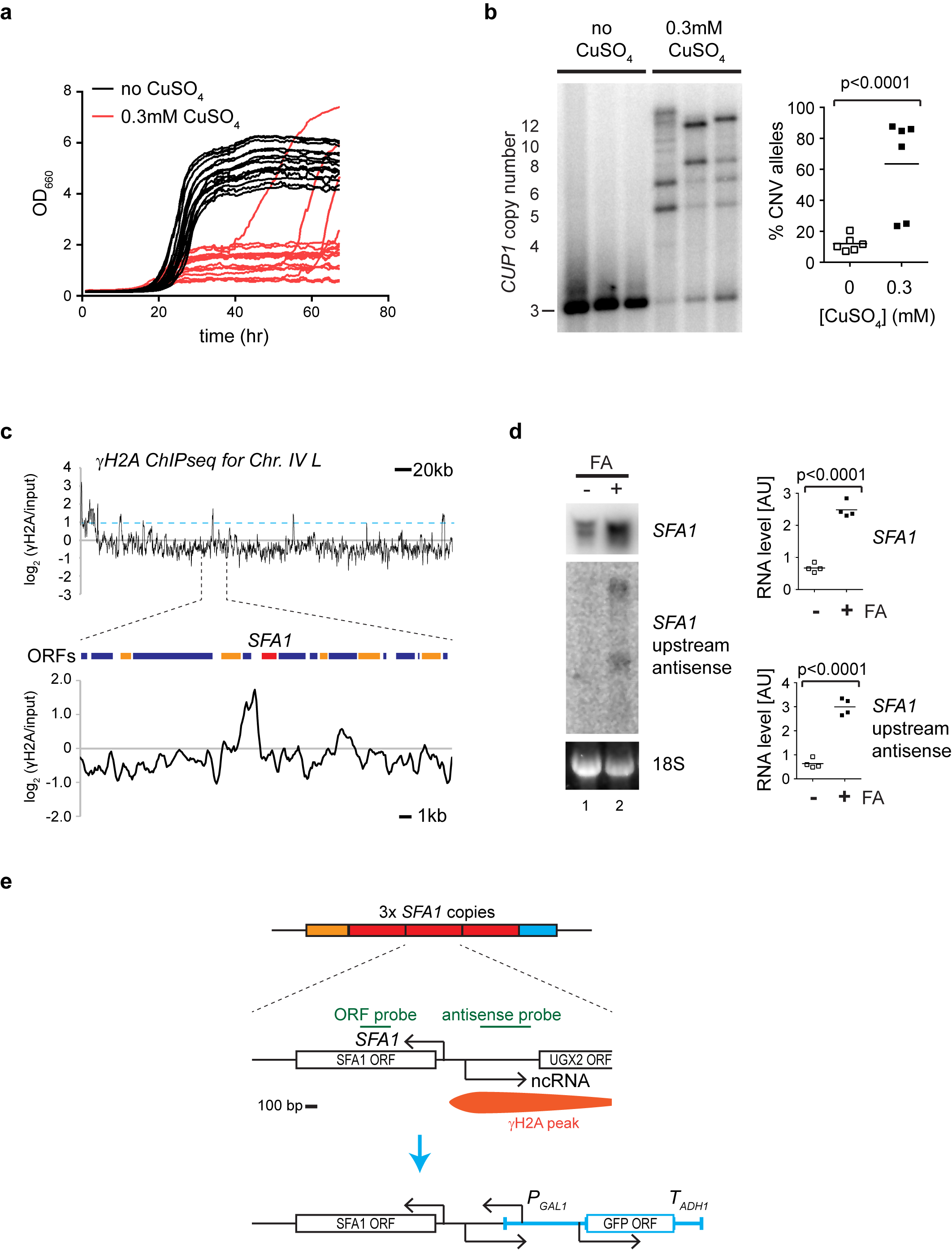

Supplement: S6 Fig — a: Growth curves of 3xCUP1 cells growing with or without 0.3 mM CuSO4. Note that the growth retardation caused by 0.3 mM CuSO4 is stronger in the 200 μl 96-well plate cultures used for growth curve analysis than in the 4 ml batch cultures used for Southern blot samples; although cells also grow slowly in 0.3 mM CuSO4 under these conditions, almost all cultures reach saturation by 72 hours. b: Southern analysis of CUP1 copy number in 3xCUP1 cells grown for 10 generations with or without 0.3 mM CuSO4. Quantification shows the percentage of amplified alleles; n = 6, p-value calculated by t test. c: γH2A signal in the region surrounding SFA1; analysis performed as in Fig 2d. d: Induction of SFA1 and upstream antisense transcripts after a 4-hour exposure to 1 mM formaldehyde, assayed by northern blot. 18S rRNA is shown as a loading control. Quantification shows the levels of the indicated RNA species in arbitrary units; p-values were calculated by t test, n = 4. Locations of probes within the SFA1 repeat are shown in e. e: Schematic of the wild-type SFA1 region in the 3xSFA1 construct, along with the modified PGAL1-GFP-SFA1 construct. All SFA1 copies carry this construct in the PGAL1-GFP-SFA1 strain. Transcriptional start sites indicated by black arrows are approximate. Raw quantitation data are available in S3, S4, S5 and S8 Data. (TIF) [file pbio.2001333.s006.tif]

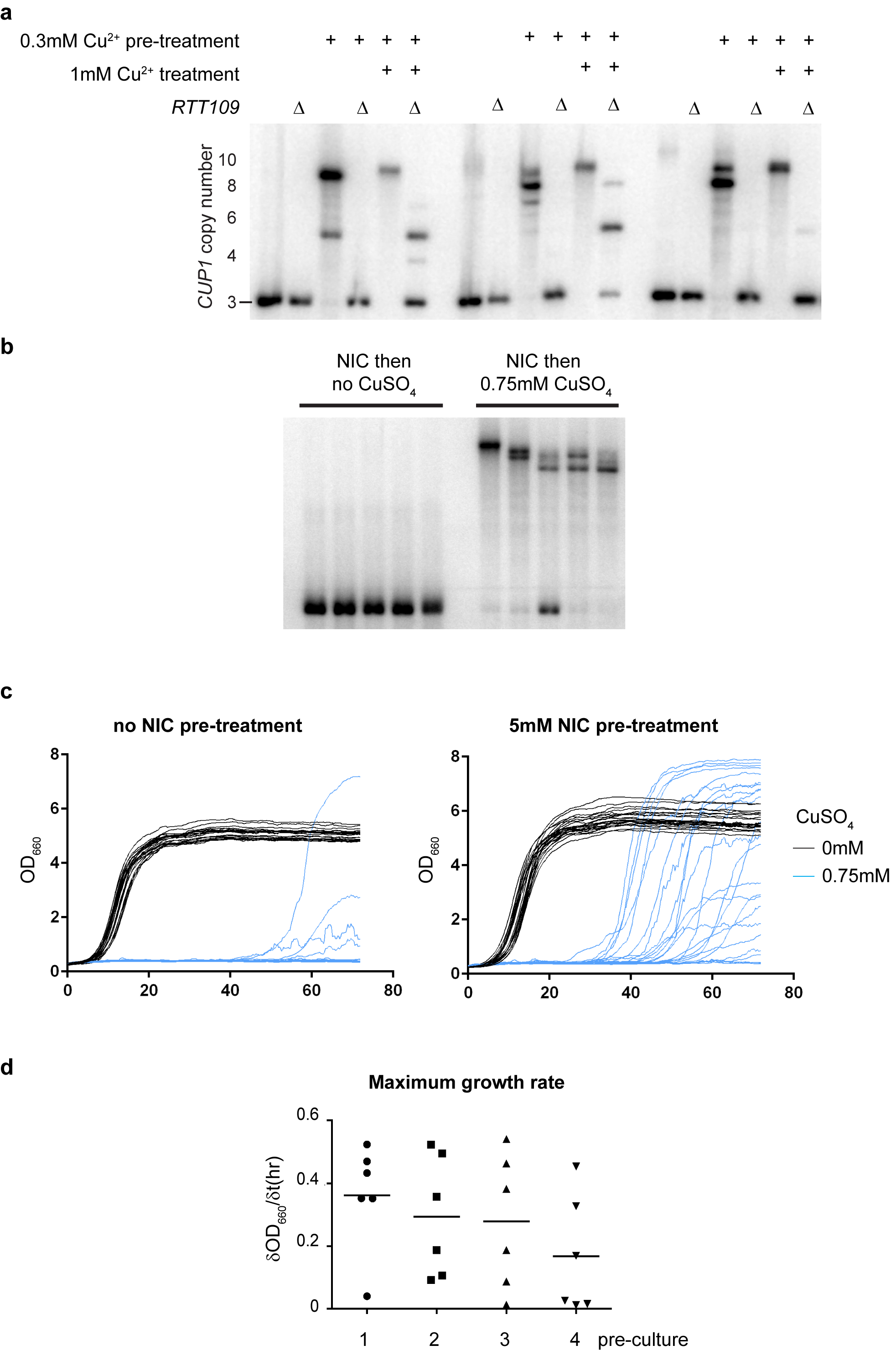

Supplement: S7 Fig — a: CUP1 copy number distribution of 3xCUP1 wild-type and rtt109Δ cells after growth for 10 generations with with or without 0.3 mM CuSO4, followed by growth with or without 1 mM CuSO4 under adaptation curve conditions, then outgrown without drug. b: CUP1 copy number distribution of 3xCUP1 cells after growth for 10 generations with 5 mM nicotinamide, followed by growth with or without 0.75 mM CuSO4 under adaptation curve conditions, then outgrown without drug. c: Individual growth curves of 3xCUP1 cells preexposed to 5 mM nicotinamide before growth with or without 0.75 mM CuSO4. d: Maximum growth rate data for +NIC +CuSO4 samples from Fig 7d, separated to show distributions of data points derived from the 4 different precultures. Raw quantitation data are available in S8 Data. (TIF) [file pbio.2001333.s007.tif]
