# Supplementary material for: Environmental change drives accelerated adaptation through stimulated copy number variation
Source: PLoS Biol. 2017 Jun 27;15(6):e2001333. doi: 10.1371/journal.pbio.2001333 (PMC5486974; doi:10.1371/journal.pbio.2001333)
Supplement: S2 Table — (DOCX) [file pbio.2001333.s009.docx]

| ID | Name | Genotype | Ref. |
| --- | --- | --- | --- |
| YRH21 | BY4741 | MATa *his3*Δ1 *leu2*Δ0 *met15*Δ0 *ura3*Δ0 | [1] |
| YJH108 | *rrp6*Δ | MATa *his3*Δ1 *leu2*Δ0 *met15*Δ0 *ura3*Δ0 *rrp6*::NatMX6 | [2] |
| YCJ3 | *hst3*Δ *hst4Δ* | MATa *his3*Δ1 *leu2*Δ0 *met15*Δ0 *ura3*Δ0 *hst3*::KanMX6 *hst4*::*LEU2* | [3] |
| YJH87 | *sir2*Δ | MATa *his3*Δ1 *leu2*Δ0 *met15*Δ0 *ura3*Δ0 *sir2*::HygMX6 | [4] |
| YJH237 | *rtt109*Δ | MATa *his3*Δ1 *leu2*Δ0 *met15*Δ0 *ura3*Δ0 *rtt109*::*KanMX4* | [5] |
| YJH243 | *mrc1*Δ | MATa *his3*Δ1 *leu2*Δ0 *met15*Δ0 *ura3*Δ0 *mrc1*::*KanMX4* | [5] |
| YJH363 | *pol32*Δ | MATa *his3*Δ1 *leu2*Δ0 *met15*Δ0 *ura3*Δ0 *pol32*::*KanMX4* | [5] |
| YJH893 | *P_GAL1_-HA* | MATa *his3*Δ1 *leu2*Δ0 *met15*Δ0 *ura3*Δ0 *ade2*::*MET25* *cup1*::17x[*P_GAL1_-3HA*]-*ADE2 pRS316-CUP1*  Note: All chromosomal *CUP1* repeats replaced with 17 copies of *CUP1* repeat in which the *CUP1* promoter and ORF are replaced by *P_GAL1_-3HA* construct. A single *ADE2* marker is present at the telomere proximal end. | This study |
| YRH82 | *P_GAL1_-HA rtt109*Δ | MATa *his3*Δ1 *leu2*Δ0 *met15*Δ0 *ura3*Δ0 *ade2*::*MET25* *cup1*::17x[*P_GAL1_-3HA*]-*ADE2 pRS316-CUP1 rtt109::KanMX4* | This study |
| YJH935 | MEP wildtype diploid | *ade2::hisG his3 leu2 lys2/+ met15::ADE2/+ ura3Δ0 trp1Δ63 hoD::SCW11pr-Cre-EBD78-NatMX loxP-UBC9-loxP-LEU2 loxP-CDC20-Intron-loxP-HPHMX* | [6] |
| YRH35 | MEP *P_GAL1_-3HA* heterozygote | *ade2::hisG/ade2::MET15 met15::ADE2/met15*Δ*0 trp1*Δ*63 his3 leu2 ura3*Δ*0 hoD::SCW11pr-Cre-EBD78-KanMX/hoD::SCW11pr-Cre-EBD78-NatMX loxP-UBC9-loxP-LEU2 loxP-CDC20-Intron-loxP-HPHMX cup1::15xPgal-HA-ADE2/CUP1* | This study |
| YJH1005 | MEP *rtt109*Δ | *ade2::hisG his3 leu2 lys2/+ met15::ADE2/+ ura3Δ0 trp1Δ63 hoD::SCW11pr-Cre-EBD78-NatMX loxP-UBC9-loxP-LEU2 loxP-CDC20-Intron-loxP-HPHMX rtt109*::*TRP1* | This study |
| YRH23/24 | 3x*CUP1* | *MATα his3Δ1 leu2Δ0 lys2Δ0 ura3Δ0 ade2Δ::LEU2 cup1Δ::ADE2-3xCUP1* | This study |
| YRH42 | 3x*CUP1 rtt109*Δ | *his3Δ1 leu2Δ0 lys2Δ0 ura3Δ0 rtt109Δ:kan ade2Δ::Leu2 cup1Δ::Ade2-3xCUP1* | This study |
| YRH89 | 3x*SFA1* | *MATα his3Δ1 leu2Δ0 lys2Δ0 ura3Δ0 ade2Δ::LEU2 cup1Δ::ADE2-1xCUP1-3xSFA1 sfa1::NatMX6* | This study |
| YRH108/109/110 | 3x*CUP1 trp1Δ* | *MATα his3Δ1 leu2Δ0 lys2Δ0 ura3Δ0 ade2Δ::Leu2 cup1Δ::Ade2-3xCUP1 trp1Δ::NatMX6* | This study |
| YRh113 | *P_GAL1_-GFP-SFA1* High-copy-number | *MATα his3Δ1 leu2Δ0 lys2Δ0 ura3Δ0 ade2Δ::Leu2 cup1Δ::1xCUP1 11x ugx2::Pgal-GFP-SFA1 sfa1::NatMX6* | This study |
| YRH100 | 3x*SFA1 rtt109*Δ | *MATα his3Δ1 leu2Δ0 lys2Δ0 ura3Δ0 ade2Δ::LEU2 cup1Δ::ADE2-1xCUP1-3xSFA1 sfa1::NatMX6 rtt109::KanMX4* | This study |

**Reference**

1. Brachmann CB, Davies A, Cost GJ, Caputo E, Li J, Hieter P, et al. Designer deletion strains derived from Saccharomyces cerevisiae S288C: a useful set of strains and plasmids for PCR-mediated gene disruption and other applications. Yeast. 1998;14(2):115-32. PubMed PMID: 9483801.

2. Houseley J, Tollervey D. Yeast Trf5p is a nuclear poly(A) polymerase. EMBO Rep. 2006;7(2):205-11. PubMed PMID: 16374505.

3. Jack CV, Cruz C, Hull RM, Keller MA, Ralser M, Houseley J. Regulation of ribosomal DNA amplification by the TOR pathway. PNAS. 2015;112(31):9674-9. doi: 10.1073/pnas.1505015112. PubMed PMID: 26195783; PubMed Central PMCID: PMC4534215.

4. Houseley J, Kotovic K, El Hage A, Tollervey D. Trf4 targets ncRNAs from telomeric and rDNA spacer regions and functions in rDNA copy number control. EMBO J. 2007;26(24):4996-5006. Epub 2007/11/17. doi: 10.1038/sj.emboj.7601921. PubMed PMID: 18007593; PubMed Central PMCID: PMC2080816.

5. Giaever G, Chu AM, Ni L, Connelly C, Riles L, Veronneau S, et al. Functional profiling of the Saccharomyces cerevisiae genome. Nature. 2002;418(6896):387-91. PubMed PMID: 12140549.

6. Lindstrom DL, Gottschling DE. The mother enrichment program: a genetic system for facile replicative life span analysis in Saccharomyces cerevisiae. Genetics. 2009;183(2):413-22, 1SI-13SI. doi: 10.1534/genetics.109.106229. PubMed PMID: 19652178; PubMed Central PMCID: PMC2766306.
