# Supplementary material for: Environmental change drives accelerated adaptation through stimulated copy number variation
Source: PLoS Biol. 2017 Jun 27;15(6):e2001333. doi: 10.1371/journal.pbio.2001333 (PMC5486974; doi:10.1371/journal.pbio.2001333)
Supplement: S3 Table — See S4 Table for oligonucleotides used to amplify cloning fragments. (DOCX) [file pbio.2001333.s010.docx]

| ID | Name | Construction |
| --- | --- | --- |
| pJH270 | pFA6a-*MET25* | *MET25* PCR cloned into pFA6a-KanMX6 backbone by *Pme*I *Asc*I. NB: This works as an auxotrophic marker but does not fully complement *met25*Δ phenotypes. |
| pJH252 | pRS316-*CUP1* | *CUP1* PCR cloned into pRS316 by *Cla*I *Not*I |
| pJH254 | pBS-*CUP1* | *CUP1* PCR cloned into pBS II SK- by *Eco*RI *Cla*I |
| pJH278 | pBS-P_GAL1_-*3HA* | Multi-fragment construction:  pBS-CUP1 *Spe*I *Eco*RI  *P_GAL1_* PCR *Spe*I *Pac*I  *3HA* PCR *Pac*I *Nhe*I  *CUP1* PCR 3’ *Nhe*I *Eco*RI |
| pJH264 | p*ADE2*-*CUP1*-flanks | Multi-fragment construction:  pBSII SK- *Kpn*I *Sac*I  *CUP1* flank PCR *Kpn*I *Cla*I  *ADE2* PCR *Cla*I *Spe*I  *CUP1* flank PCR *Spe*I *Sac*I |
| pJH280 | p*ADE2* 3x P_GAL1_-*3HA* | Multi-fragment construction:  pADE2-*CUP1*-flanks *Cla*I *Eco*RI  pBS-P_GAL1_-3HA *Cla*I *Sal*I  pBS-P_GAL1_-3HA *Xho*I *Ngo*MIV  pBS-P_GAL1_-3HA *Xma*I *Eco*RI |
| pRH9 | p*ADE2* *CUP1* | Multi-fragment construction:  pBS II SK- *Xho*I *Sac*I  pADE2-*CUP1*-Flanks *Eco*RI *Sac*I  *RSC30* right flank PCR *Xho*I *Eco*RI |
| pRH12 | p*ADE2* 3x*CUP1* | Multi-fragment construction:  p*ADE2 CUP1 Sal1 EcoR1*  pBS-*CUP1 Xho1 BglII*  pBS-*CUP1 BamH1 EcoR1* |
| pJH294 | pBS *SFA1* | *SFA1* PCR cloned into pBS II SK- by *Xho*I *Not*I |
| pJH310 | p*ADE2*-*CUP1*-1x*SFA1* | *Xho*I *Eco*RI fragment of pJH294 cloned into *Sal*I *Eco*RI sites of pRH9 |
| pJH311 | pBS 2x*SFA1* | *Xho*I *Eco*RI fragment of pJH294 cloned into *Sal*I *Eco*RI sites of pJH294 |
| pJH312 | p*ADE2*-*CUP1*-3x*SFA1* | *Xho*I *Eco*RI fragment of pJH311 cloned into *Sal*I *Eco*RI sites of pJH310 |
| pJH337 | pGEM SFA1 part | PCR 1460-1234 on BY4741 gDNA in pGEM-Teasy |
| pJH338 | pJET Pgal-GFP | Multi-fragment construction:  PCR 1485-1110 on BY4741 gDNA *Pac*I digest  PCR pFA6a F1-1486 on pFA6a-GFP-TRP1 *Pac*I digest  pJET EcoRV |
| pJH340 | pGEM *ugx2::Pgal-GFP SFA1* | pJH337 *Nhe*I *Pst*I + pJH338 *Nhe*I *Pst*I |
| pJH347 | pGEM 2x  *ugx2::Pgal-GFP SFA1* | pJH340 *Xho*I *Xma*I + pJH340 *Xho*I *Ngo*MIV |
| pJH348 | p*ADE2* *CUP1 ugx2::Pgal-GFP SFA1* | pRH9 *Sal*I *Eco*RI + pJH340 *Xho*I *Eco*RI |
| pJH350 | p*ADE2* *CUP1 3x ugx2::Pgal-GFP SFA1* | pJH348 *Sal*I *Eco*RI + pJH347  *Xho*I *Eco*RI |
