# Supplementary material for: Environmental change drives accelerated adaptation through stimulated copy number variation
Source: PLoS Biol. 2017 Jun 27;15(6):e2001333. doi: 10.1371/journal.pbio.2001333 (PMC5486974; doi:10.1371/journal.pbio.2001333)
Supplement: S4 Table — (DOCX) [file pbio.2001333.s011.docx]

| Oligonucleotide sequences | Description |
| --- | --- |
| AGTTTCGATAGTTTAAACTCCGACTATATCTGAGACGAACA…  TATGAAATCTGGCGCGCCTGGTGGTTGGCAAATGAC | MET25 – on BY4741 |
| ATTTTATAATTATTTGCTGTACAAGTATATCAATAAACTTATATAGAATTCGAGCTCGTTTAAAC…  AGTATATCATCTCATTTCCGTAAATACCAAATGTATTATATATTGCGGATCCCCGGGTTAATTAAG | Deletion cassette for ADE2 – on pFA6a-MET25 plasmid |
| CACTGTTACCATCGATACTCGAGGATCCCGGGATTCATGGTACCCGCTGCTGAA…  AACTGACCATGCGGCCGCGAATTCAGATCTGTCGACGCCGGCTTTTTTTTTTTTTTTTATTCGA | *CUP1* repeat for pBS *CUP1* and pRS316 *CUP1* – on BY4741 gDNA |
| TCGTATTAGTACTAGTTTATATTGAATTTTCAAAAATTCTTAC…  ATTTTGGAAGTTAATTAATTCGCTGAACATTATAGTTTTTTCTCCTTGACGTT | P_GAL1_ – on BY4741 gDNA |
| CGGATCCCCGGGTTAATTAAG…  TATGGTCTCTGCTAGCTCAGCACTGAGCAGCGTAATC | 3HA – on pFA6a-3HA-KanMX6 |
| GTGTTCAACTGCTAGCAACGAATAGTCTTTAATATATTCATCTAAC…  AACTGACCATGCGGCCGCGAATTCAGATCTGTCGACGCCGGCTTTTTTTTTTTTTTTTATTCGA | *CUP1* 3’ – on BY4741 gDNA |
| TACGCTCAGTGGTACCGAGCTCTCAATGACCCTATTCAATAAGCA…  GAACTCAGTCATCGATGAAGACTGACCTAGAAGCGAATG | *CUP1* flank – on BY4741 gDNA |
| CCATCGATTTACTAGTGCATATGTATTAATCCTAAAATGTATTAT…  TTAACTAGGAGAGCTCTAGCGAGTCAGAAGCTGTCAAG | *CUP1* flank – on BY4741 gDNA |
| TAGTGCTAGTATCGATTGAATCGAATTCCACCATAGATCTGAATTAATTCTTGA…  GGTCGATCCTACTAGTCACACCGCATAGATCTTATGTATG | *ADE2* for 3xP_GAL1_-3HA plasmid – on BY4741 |
| ATTTTACCTTTAAAAGACGTTCTCATAATACATTTTAGGATTAATACATAGAATTCGAGCTCGTTTAAAC…  TTTTTGAAAAAAATGTATTACTCAAGACATTCGCTTCTAGGTCAGTCTTCCGGATCCCCGGGTTAATTAAG | Deletion cassette for *CUP1* repeats – on pFA6a-LEU2 plasmid |
| CAGTAGAGTTAAAAGGTCAATTCAACCGGTCTTCAATAAGACATGCGGATCCCCGGGTTAATTAAG…  CGATGCTACATACGTGTACTAAATAATAAATATCAATATGTATCAGAATTCGAGCTCGTTTAAAC | Deletion cassette for *RTT109* - on pFA6a-TRP1 plasmid |
| AATTCTAAGAAAGGCAAGGTTGA…  GAACTCAGTCATCGATGAAGACTGACCTAGAAGCGAATG | *CUP1* flank probe (single copy target) – on BY4741 gDNA |
| AACTGACCATGCATGCTAGTTAGAAAAAGACATTTTTGCTGT…  GGATCCTAATACGACTCACTATAGGGAGAGGATCATTTCCCAGAGCAGCATGA | *CUP1* specific probe (multi-copy target) – on BY4741 gDNA |
| TCGTATTAGTACTAGTTTATATTGAATTTTCAAAAATTCTTAC…  GGATCCTAATACGACTCACTATAGGGAGAGGATCAGCACTGAGCAGCGTAATC | *P_GAL1_-3HA* specific probe (multi-copy target) – on pBS-P_GAL1_-3HA gDNA |
| ATGTTCAGCGAATTAATTAACTTCCA…  GGATCCTAATACGACTCACTATAGGGAGAGGATCATTTCCCAGAGCAGCATGA | *CUP1* RNA probe – on BY4741 gDNA |
| GGATCCTAATACGACTCACTATAGGGAGAGGAGCGTATCCTTTTTACGAGATGAAA…  CCGTATAAACCTATACACATATA | *CUP1* CUT RNA probe – on BY4741 gDNA |
| TGATAAATCCCCGTTAAGTCGTA… GCCAACCTGAGCAGTAGAGTAA | *rtt109::KanMX4* – on YJH237 gDNA |
| TGATTATTTTCAGGGGTGTCCGAGTCCACCTCTACAACATCCACC CGGATCCCCGGGTTAATTAAG…  TTCCAGAAAATTTGAGTCATGCTTACTTAGTTTAATTAAGTACTC GAATTCGAGCTCGTTTAAAC | *SFA1* deletion – on pFA6a-NatMX6 |
| CACTGTTACCATCGATaCTCGAGtctagaCCCGGGTGCCCGGTTTGATACCTGTA…  AACTGACCATgcggccgcGAATTCactaGTCGACGCCGGCTTTTTTTTTTTTTTTTCAAAAGTGACAAAGCTAACGT | *SFA1* cloning – on BY4741 gDNA |
| CGTATGATGCGAAGAAACCA…CCTGTGAGTGATAAATTCTTCGA | *SFA1* probe – on BY4741 gDNA |
| TACGCTCAGTCTCGAGGAGCTCTCAATGACCCTATTCAATAAGCA…  AACTGACCATgcggccgcGAATTCAGATCTGTCGACGCCGGCTTTTTTTTTTTTTTTTATTCGA | *RSC30* right flank - on BY4741 gDNA |
| CGTATGATGCGAAGAAACCA…  GGATCCTAATACGACTCACTATAGGGAGAGGA CGCCTTTTTGCACATTAGCT | *SFA1* ORF probe (southern) - on BY4741 gDNA |
| GGATCCTAATACGACTCACTATAGGGAGAGGA ATCCTTATCAAATGATACGCTCTT…  ATAGGCCAACAAATTCTGACACT | *SFA1* upstream CUT probe - on BY4741 gDNA |
| tcgtattagt gctagc TTATATTGAATTTTCAAAAATTCTTAC…  ATTTTGGAAG TTAATTAA TTCGCTGAACAT TATAGTTTTTTCTCCTTGACGTT | *P_GAL1_* for  *P_GAL1_-GFP SFA1* construct (1485-1110) - on BY4741 gDNA |
| aatcgcttca gctagc GATTTAAGGCGGTAAGAAGGA…  AACTGACCATgcggccgcGAATTCactaGTCGACGCCGGCTTTTTTTTTTTTTT TTCAAAAGTGACAAAGCTAACGT | *SFA1* for  *P_GAL1_-GFP SFA1* construct (1460-1234) - on BY4741 gDNA |
| CGGATCCCCGGGTTAATTAA…  aatcgcttca cccggg TGATCTATATTACCCTGTTATCCCTAG | *GFP* for  *P_GAL1_-GFP SFA1* construct ( pFA6a F1-1486) – on pFA6a-GFP-TRP1 |
