# Supplementary material for: Environmental change drives accelerated adaptation through stimulated copy number variation
Source: PLoS Biol. 2017 Jun 27;15(6):e2001333. doi: 10.1371/journal.pbio.2001333 (PMC5486974; doi:10.1371/journal.pbio.2001333)
Supplement: S9 Data — (PDF) [file pbio.2001333.s021.pdf]

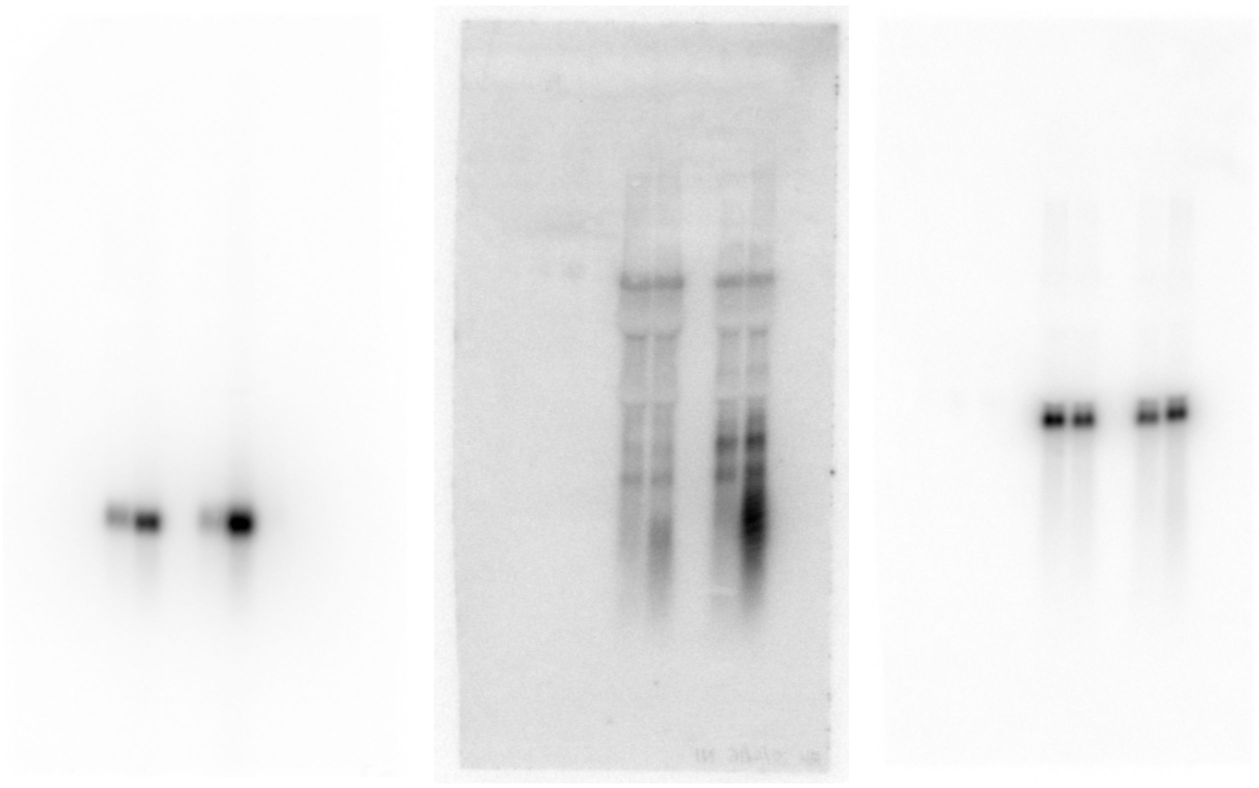

CUP1 northern blot in Fig. 2c. Two duplicate membranes of the same samples, left membrane probed for *CUP1* sense, middle membrane for *CUP1* antisense CUT then stripped and re-probed for *ACT1* (right)

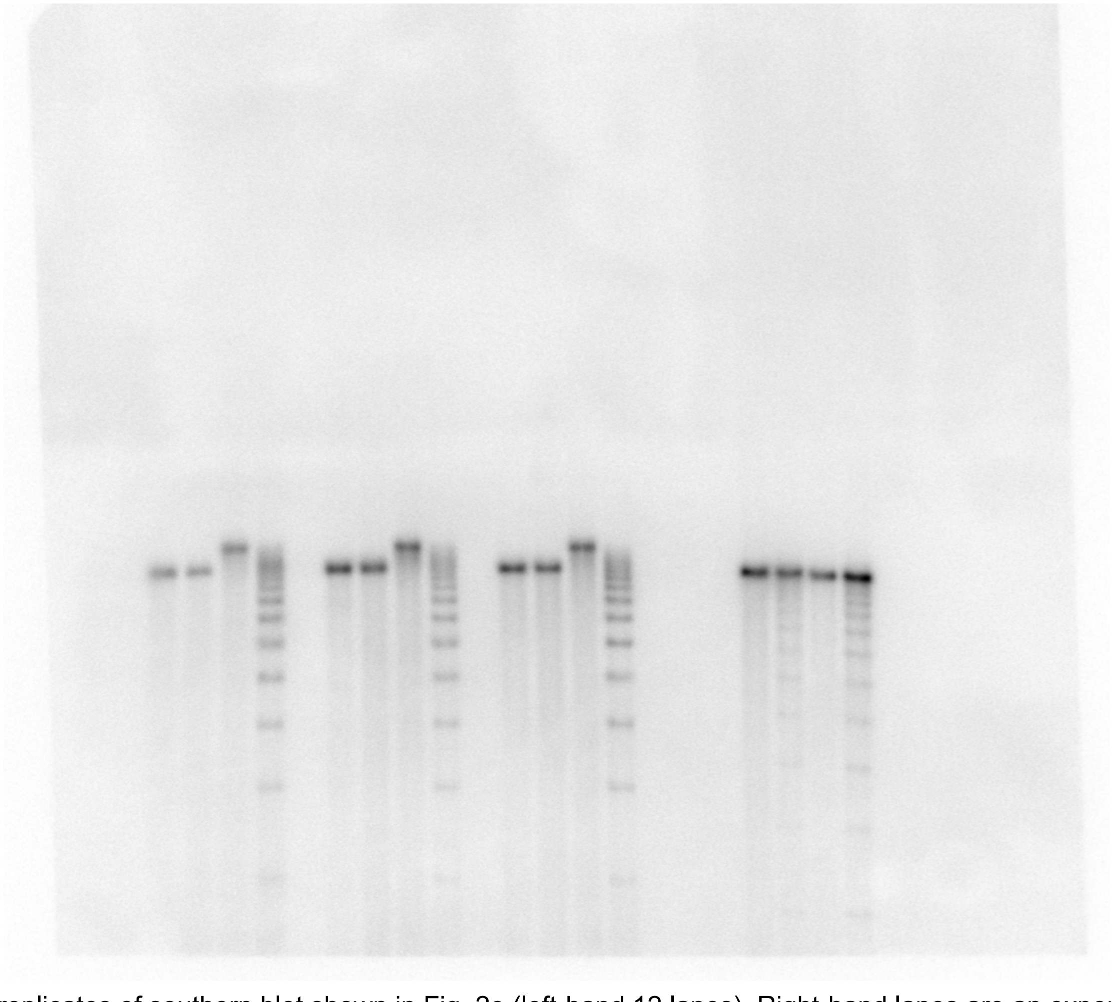

3 replicates of southern blot shown in Fig. 2e (left-hand 12 lanes). Right-hand lanes are an experiment not included in this manuscript.

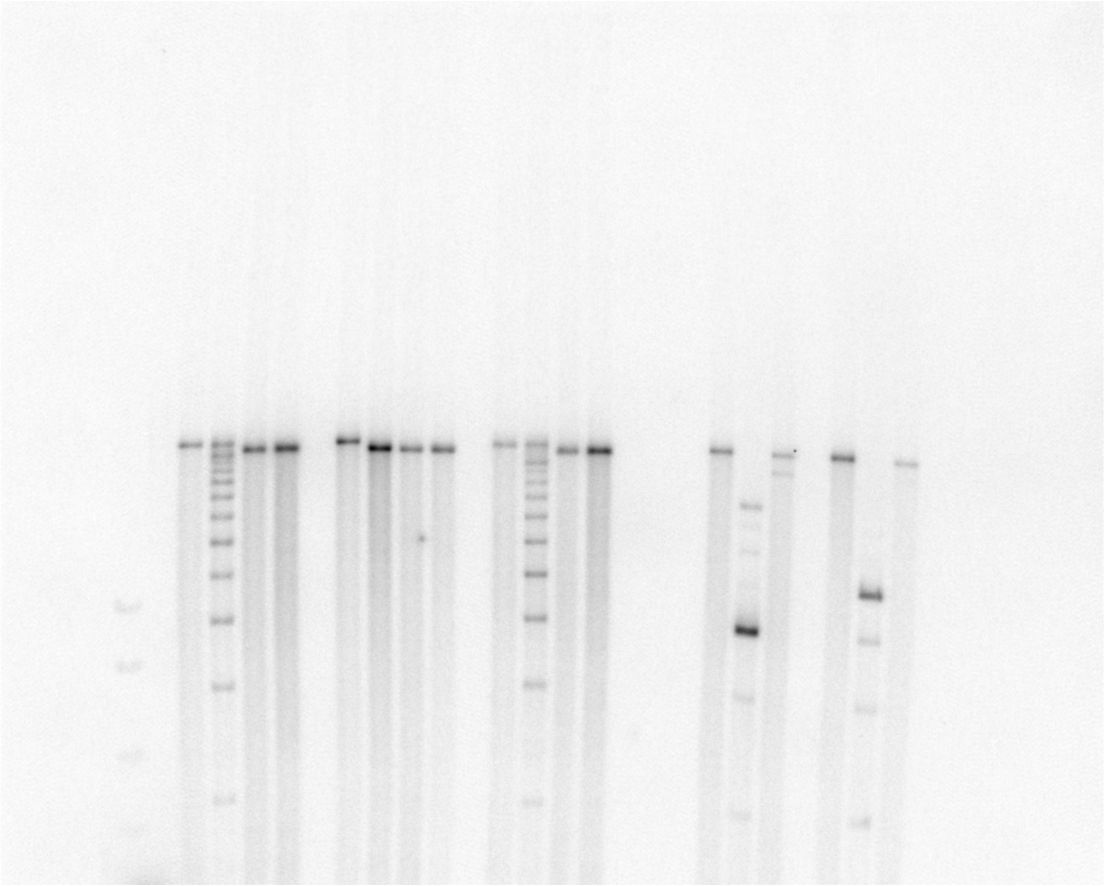

Southern blot from Fig. 4a. Two replicates - lanes 1-4 and 9-12. Lanes 5-8 contain a replicate in which an *rtt109* mutant was mistakenly innoculated in the second culture (this was confirmed by PCR genotyping of DNA samples loaded on gel). This replicate was repeated on another membrane (not shown), giving a similar result to replicates 1 and 3. Right-hand lanes are not included in this manuscript.

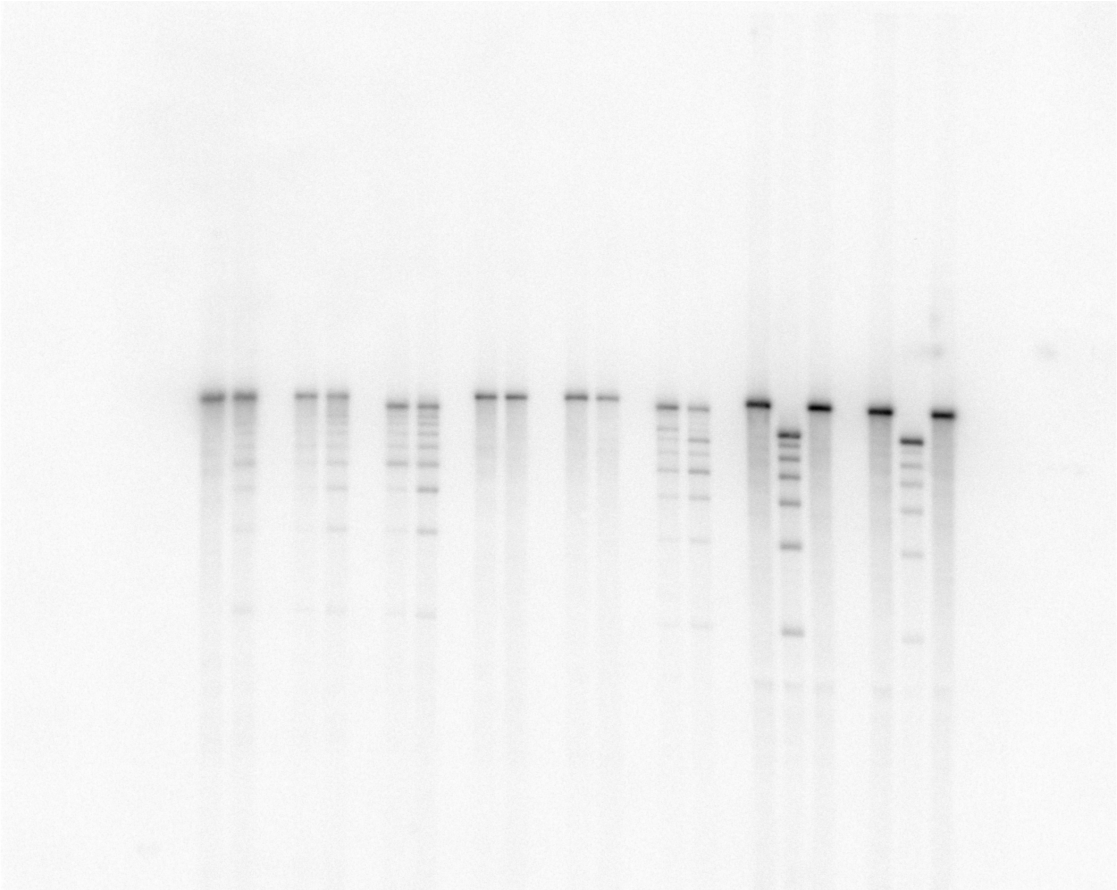

Southern blot from Fig. S3a. Lanes 13-15 ad 16-18 contain two replicates, lanes 1-12 are not included in this manuscript.

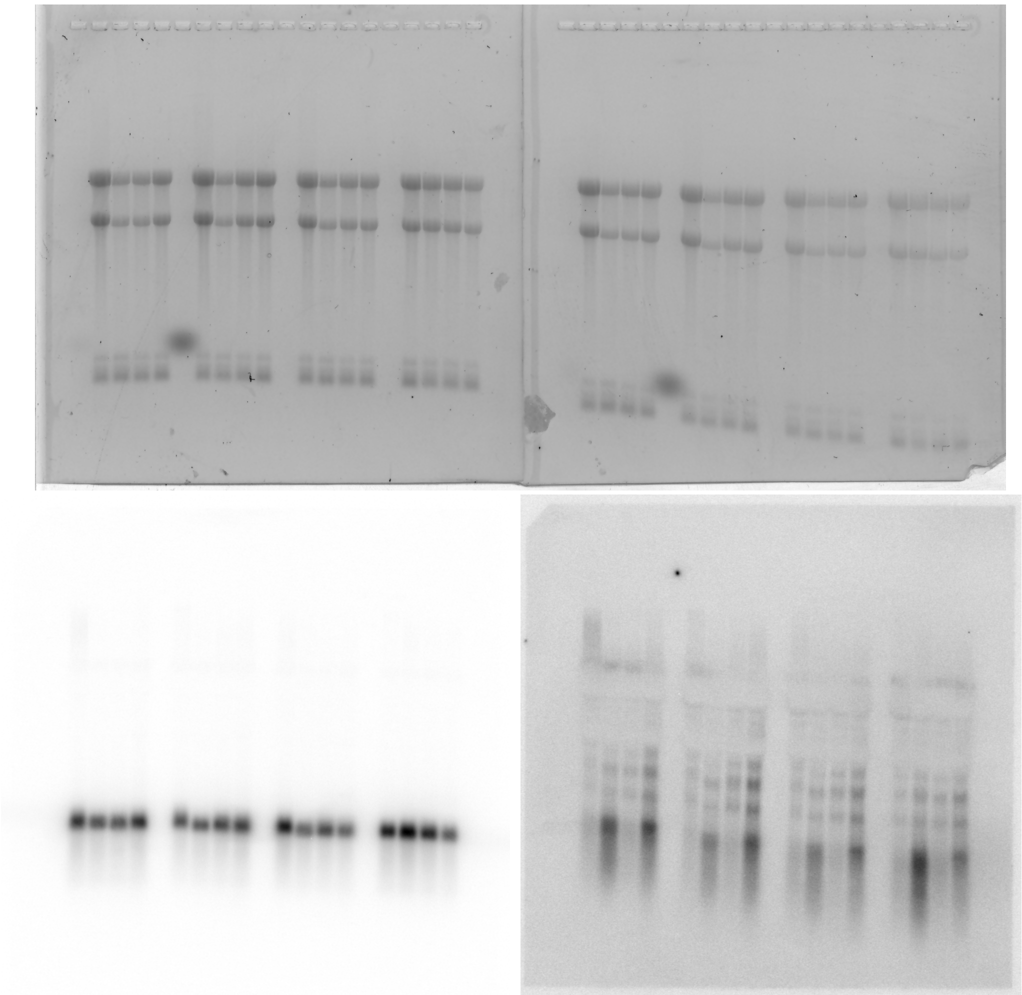

Northern blot from Fig. 4c. Samples were run on two replicate gels (ethidium bromide stained gels shown), blotted and probed for CUP1 ORF (left) and CUP1 CUT (right). Samples are organised as 4 replicates.

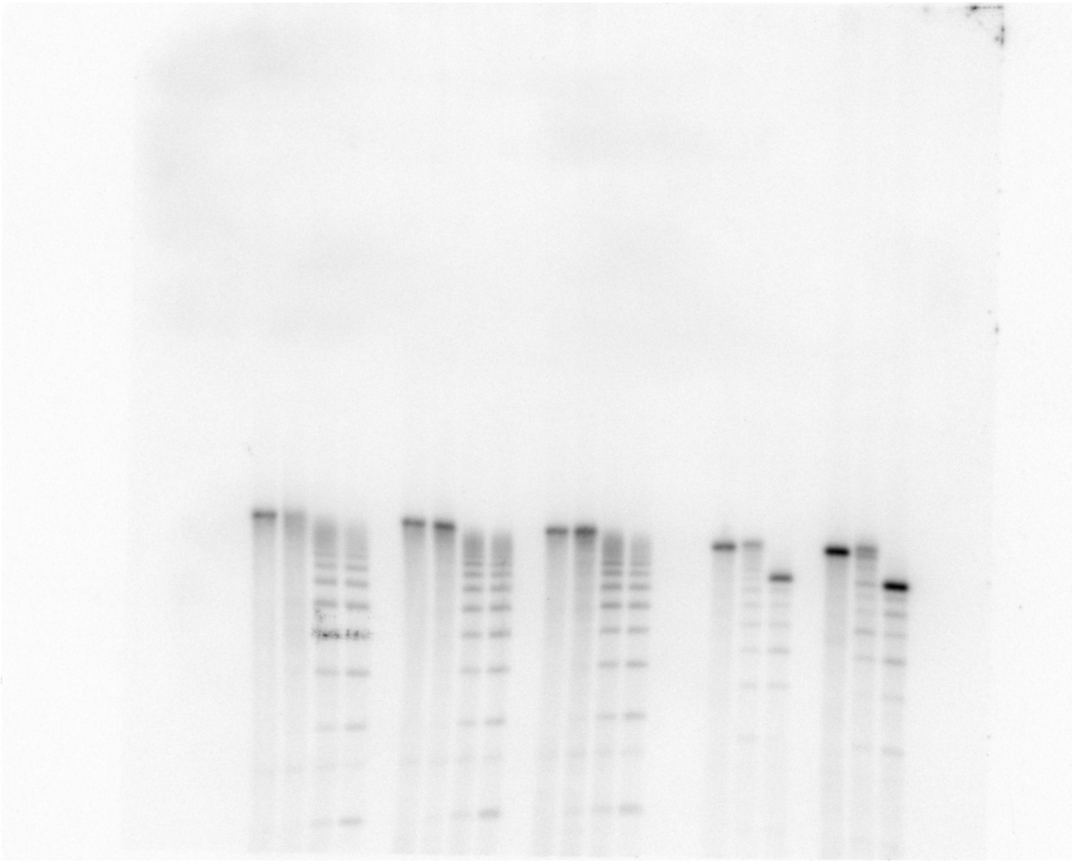

Southern blots Fig 4d and 5a. Lanes 1-12 contain 3 replicates of 5a, lanes 13-18 contain 2 replicates of 3d.

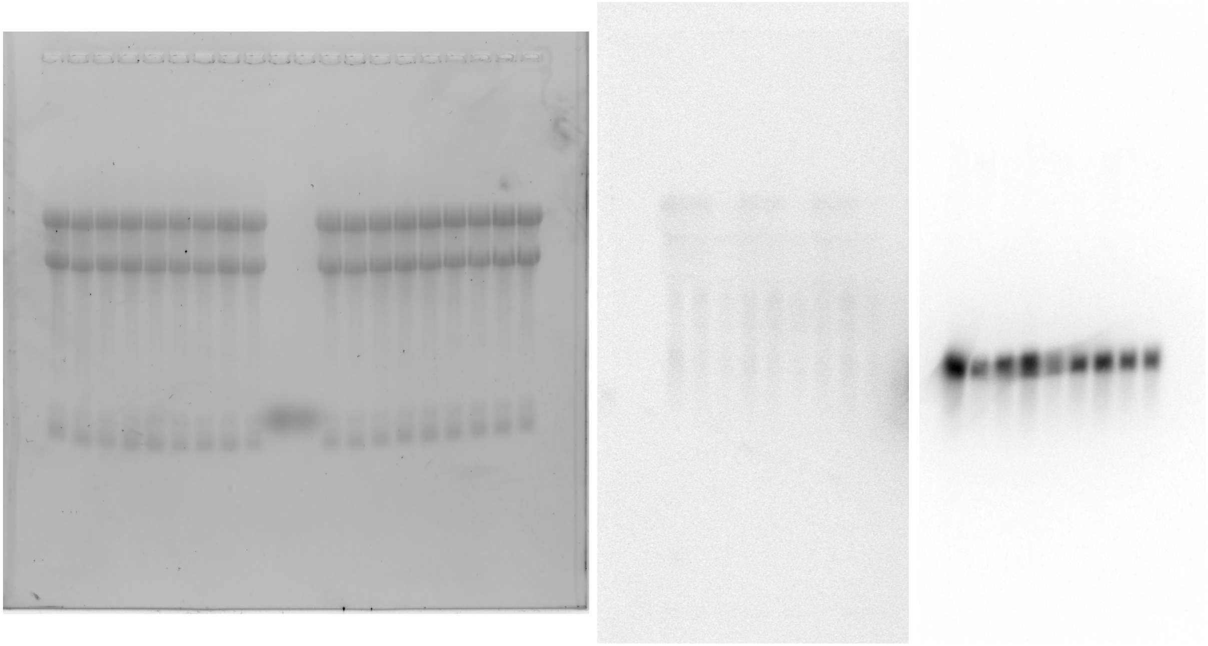

Northern blot from Fig. S3b. The same samples were separated twice on a single gel, ethidium stained then blotted, split and blots probed for CUP1 CUT (middle) or CUP1 ORF (right). Each membrane has 3 replicate sample sets.

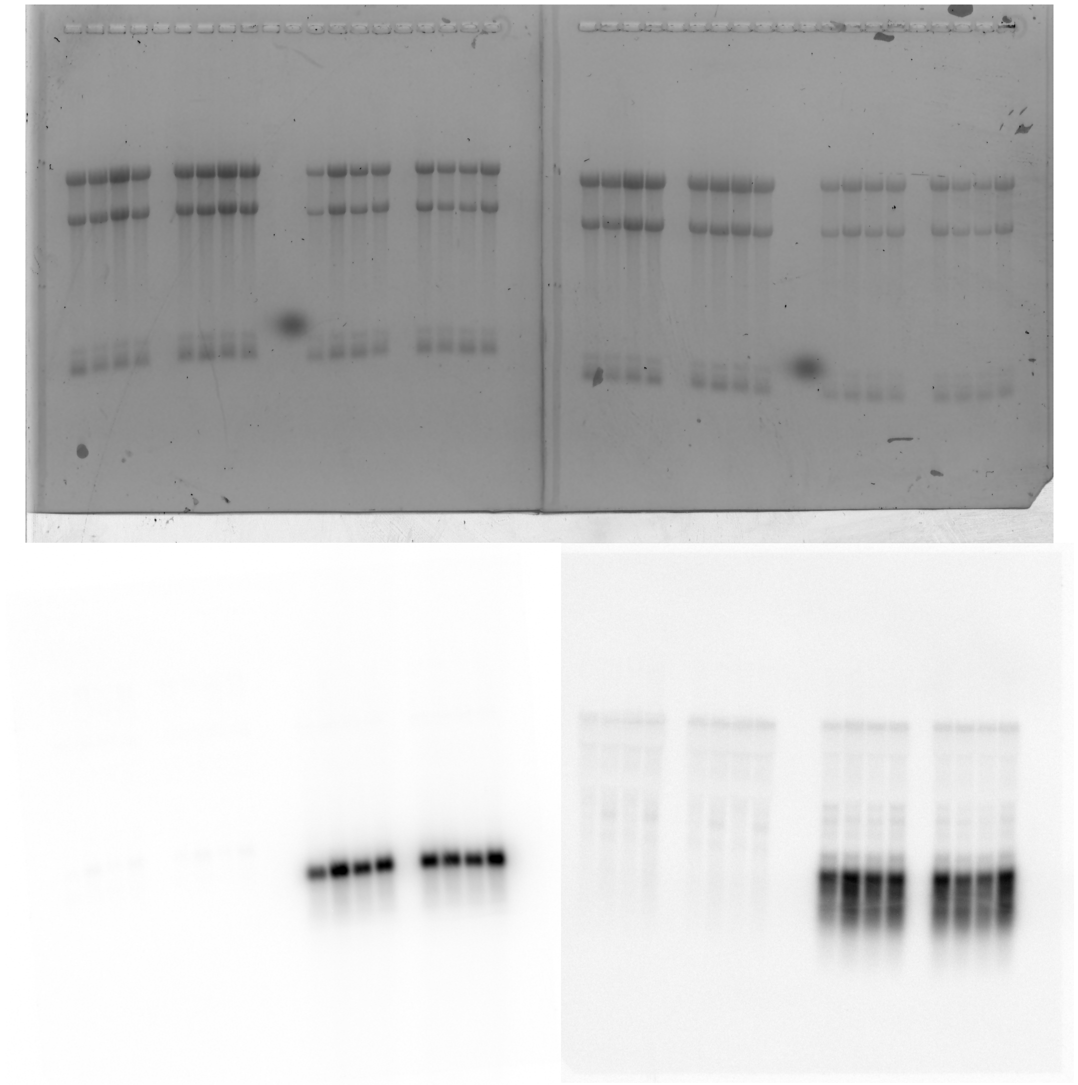

Northern blot from Fig. S4. The same samples were separated twice on two gels, ethidium stained then blotted and probed for HA ORF (left) or CUP1 CUT (right). Sample order is unusual because of high expected signal difference: 4x [GLU -/+ NIC] then 4x [GAL -/+ NIC]. Presented image is lanes 7-10.

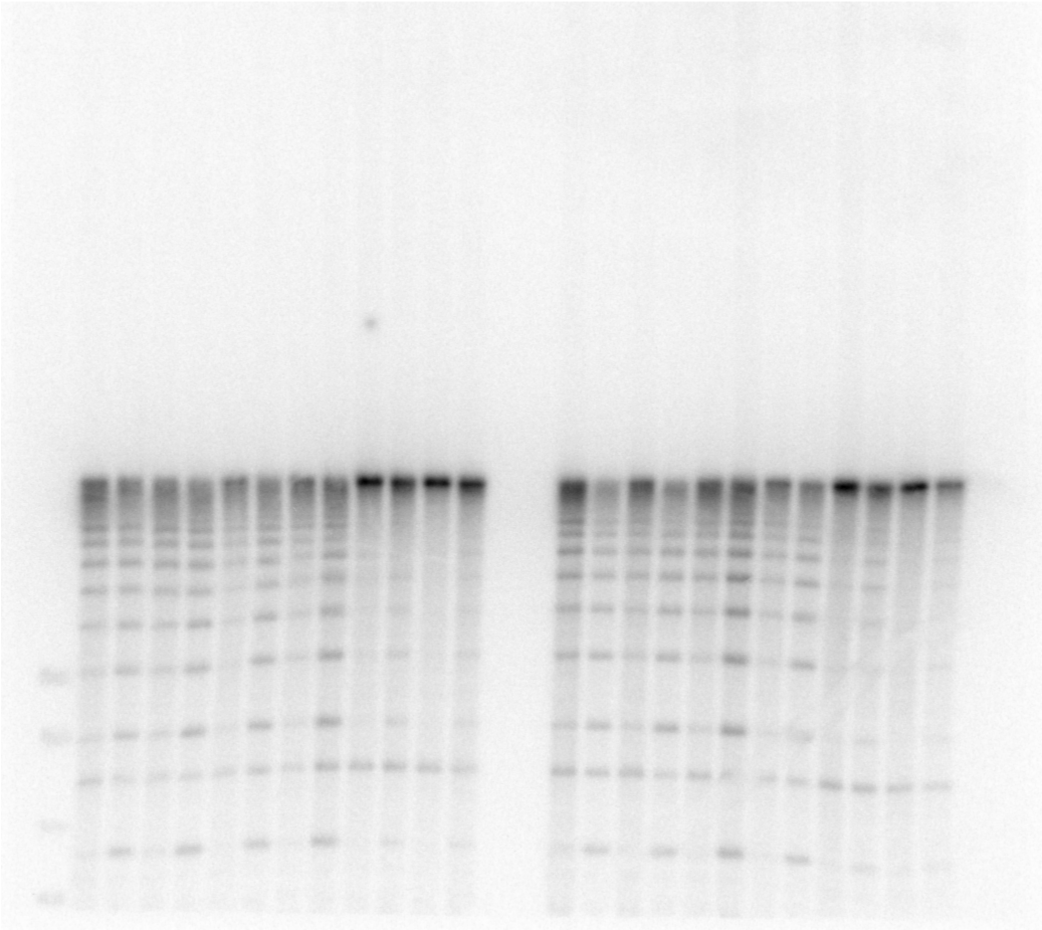

Southern blot from Fig. 5b (left 12 lanes), another replicate is shown in right-hand 12 lanes

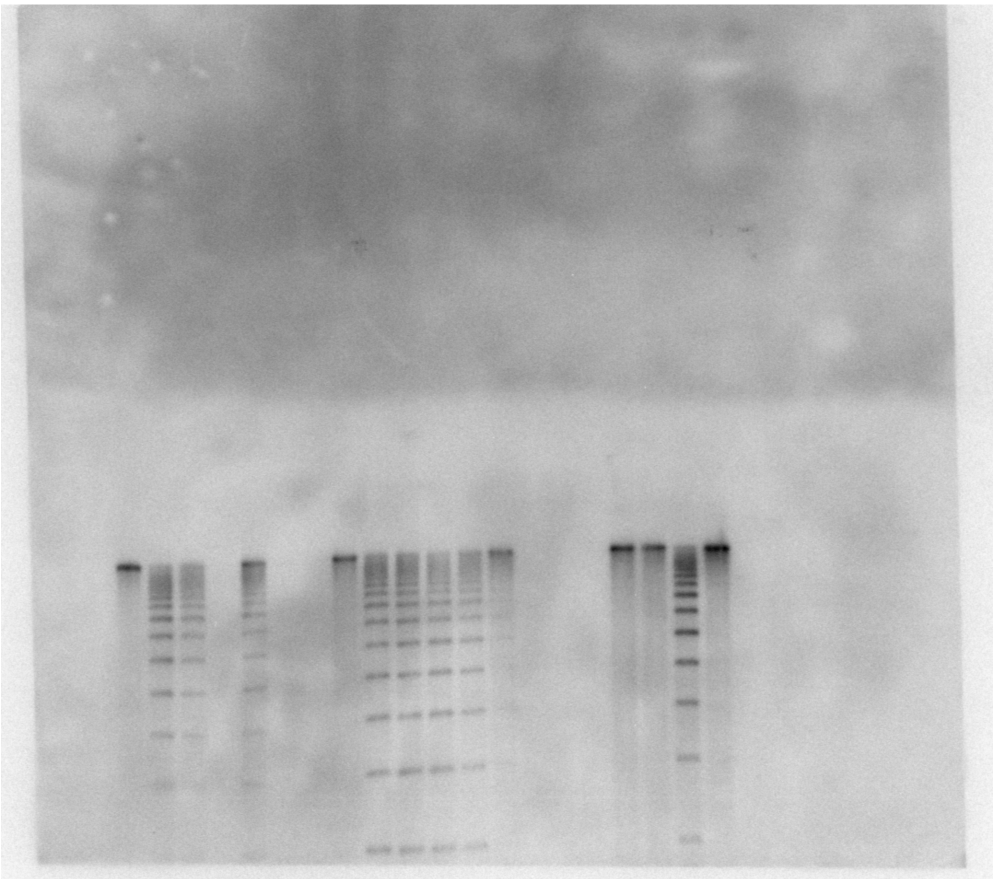

Southern blot from Fig. 5c (right-hand 4 lanes). The high background on this membrane did not impact the result.

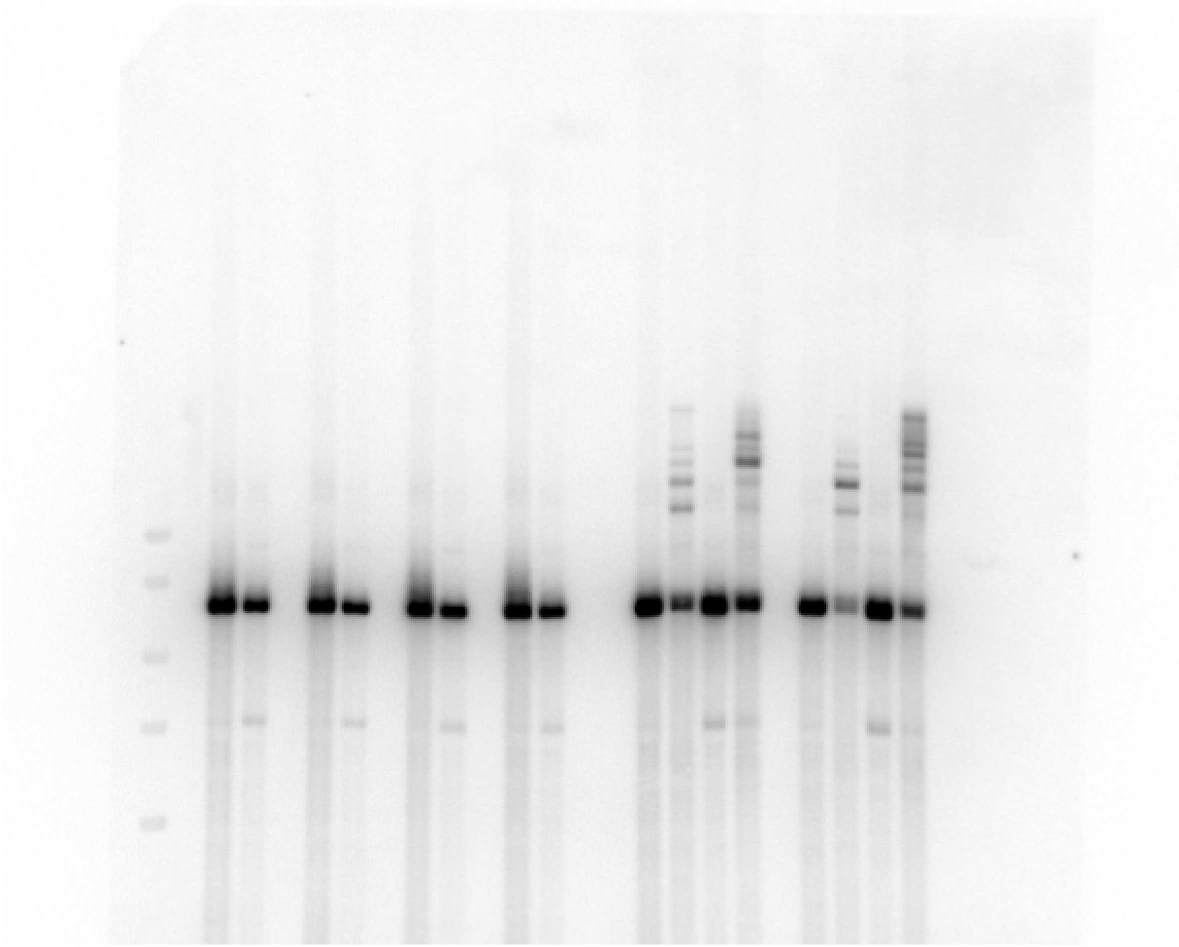

Southern blots Fig. 6b and 6c. Lanes 1-8 contain 4 replicates of 6b, lanes 9-16 contain 2 replicates of 6c.

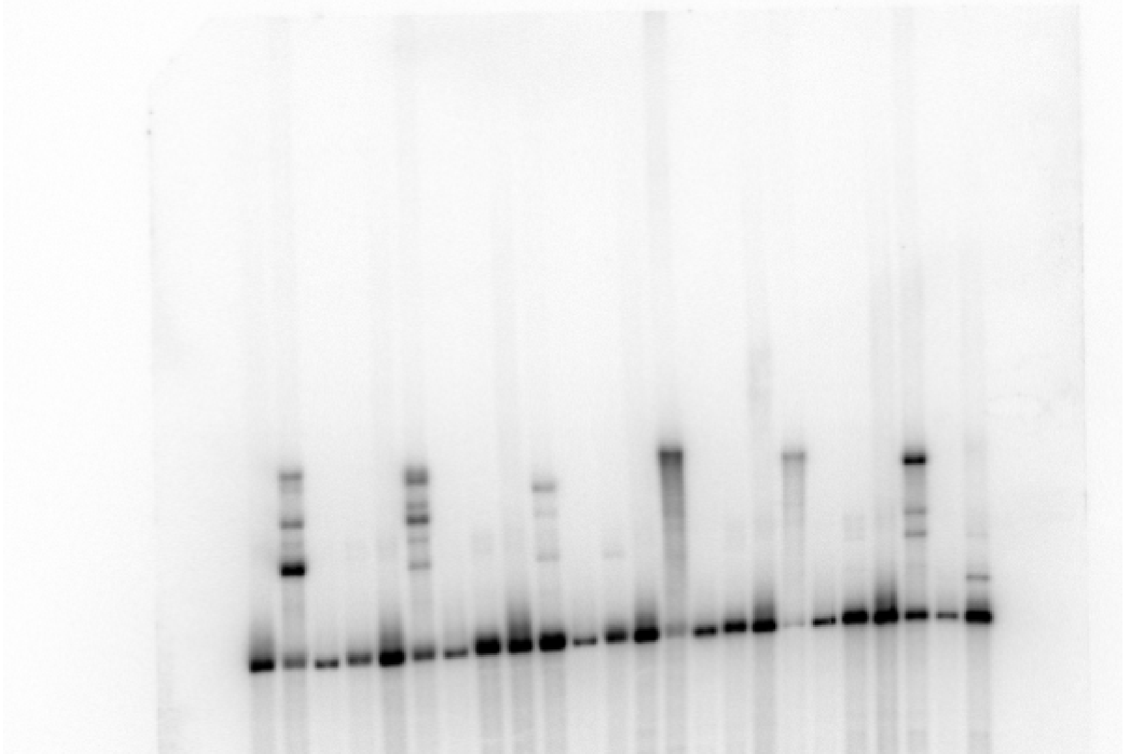

6 replicates of Southern blot shown in Fig. 6d.

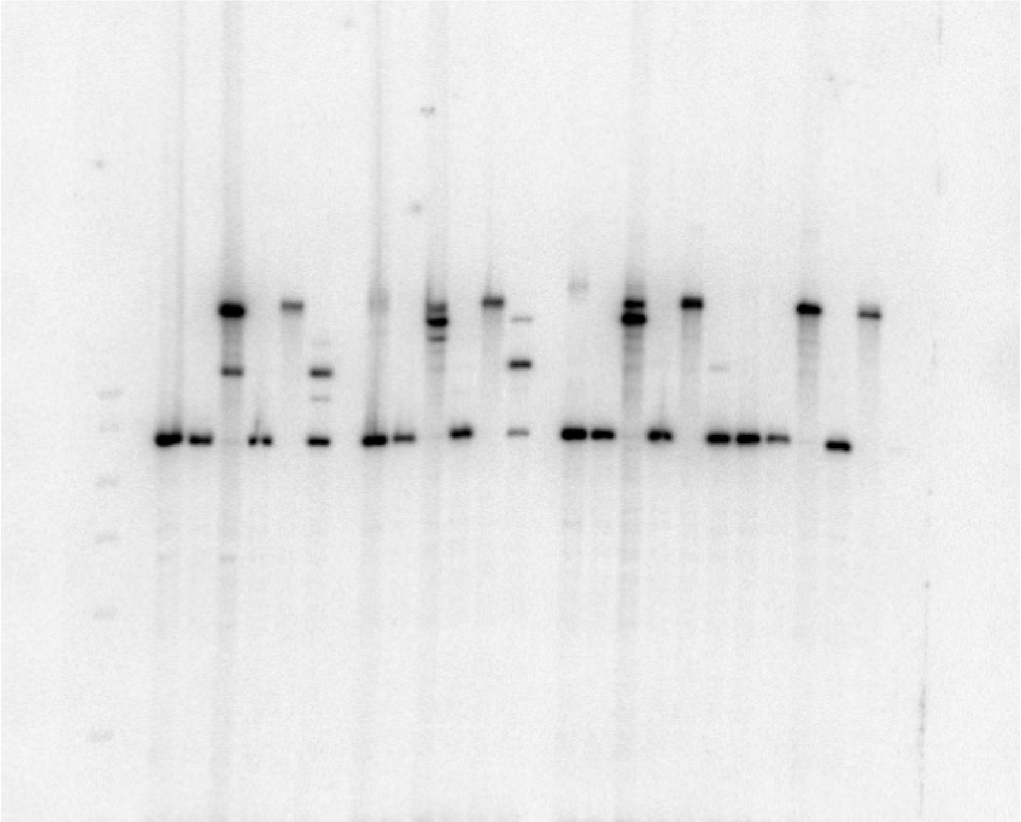

4 replicates of Southern blot shown in Fig. S7a.

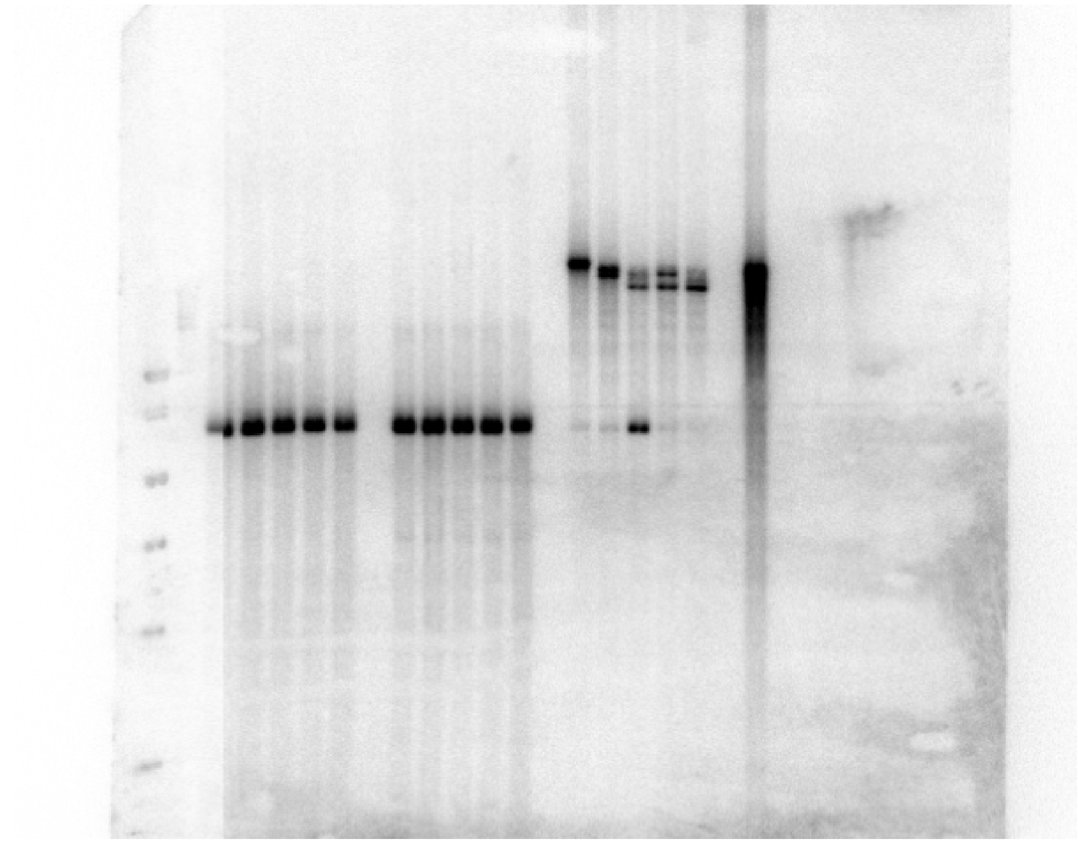

Southern blot from Fig. S7b. Lanes 6-15 contain Fig. S7b. Lanes 1-5 and 16 were not included in this manuscript.

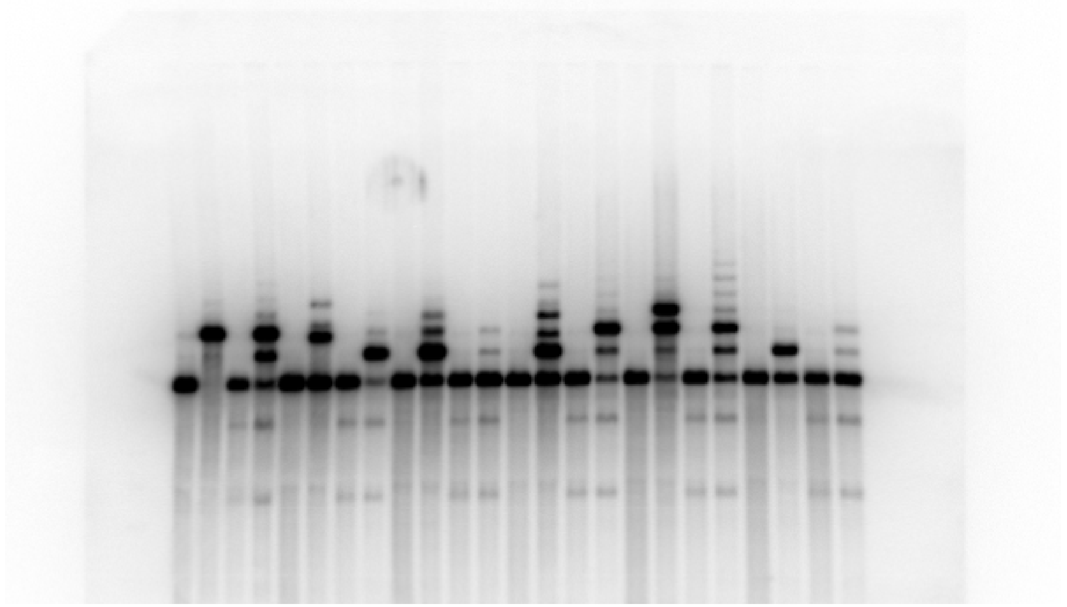

6 replicates of Southern blot shown in Fig. 6e.

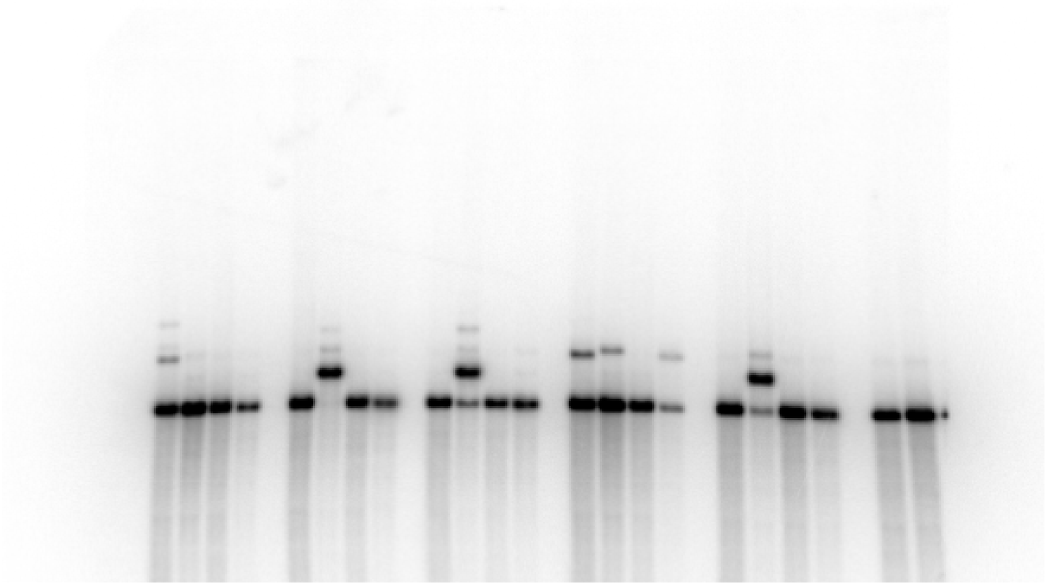

5 replicates of Southern blot shown in Fig. 6f. Right-hand two lanes come from an incomplete 6th replicate set that was not included in any of the analysis.

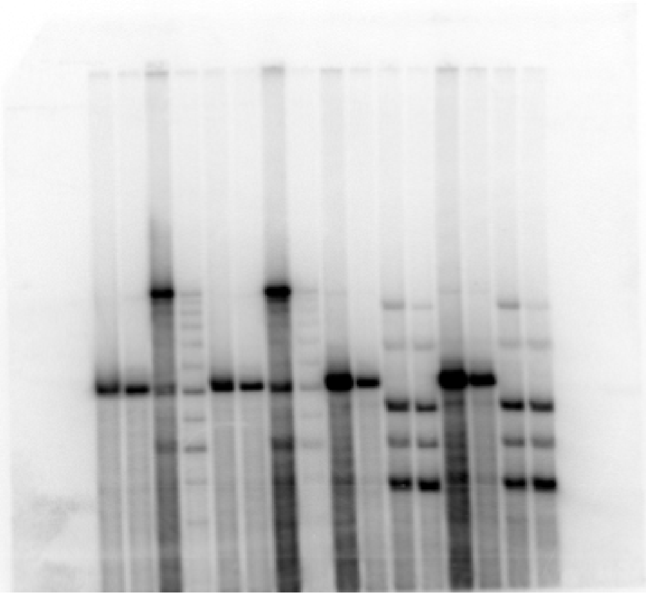

Southern blot of Fig. 6g. Lanes 1-8 contain 2 replicates of Fig. 6g. Lanes 9-16 are an experiment not included in this manuscript.

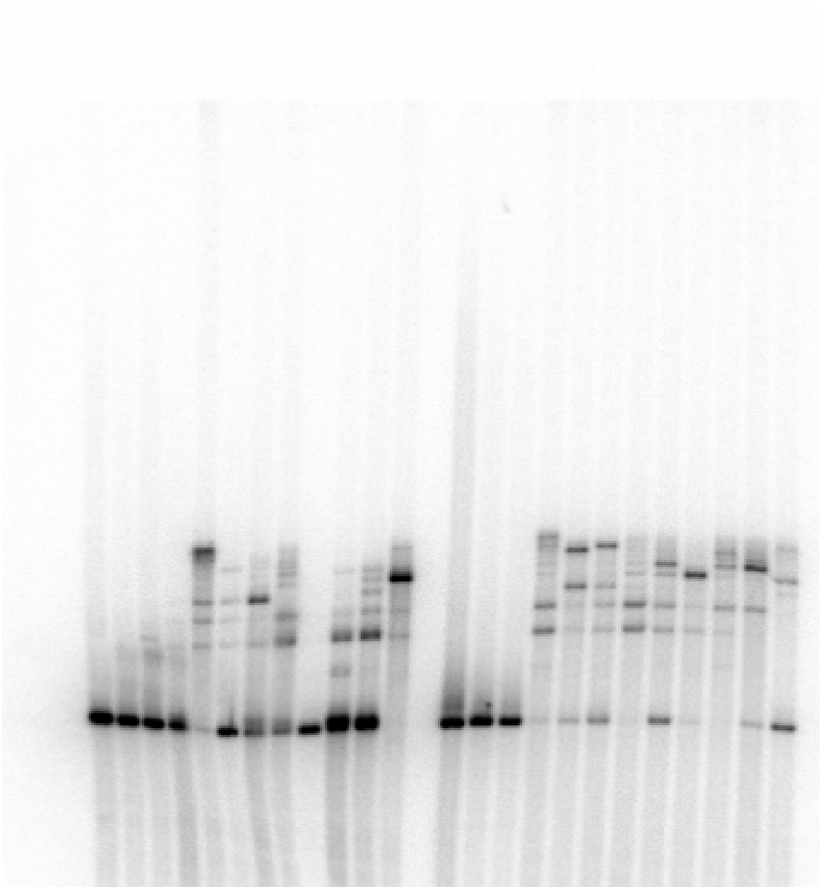

Southern blot of Fig. S6b. Lanes 1-6 and 13-18 contain 2 replicates of Fig. S6b. Lanes 7-12 and 19-24 are an experiment not included in this manuscript.

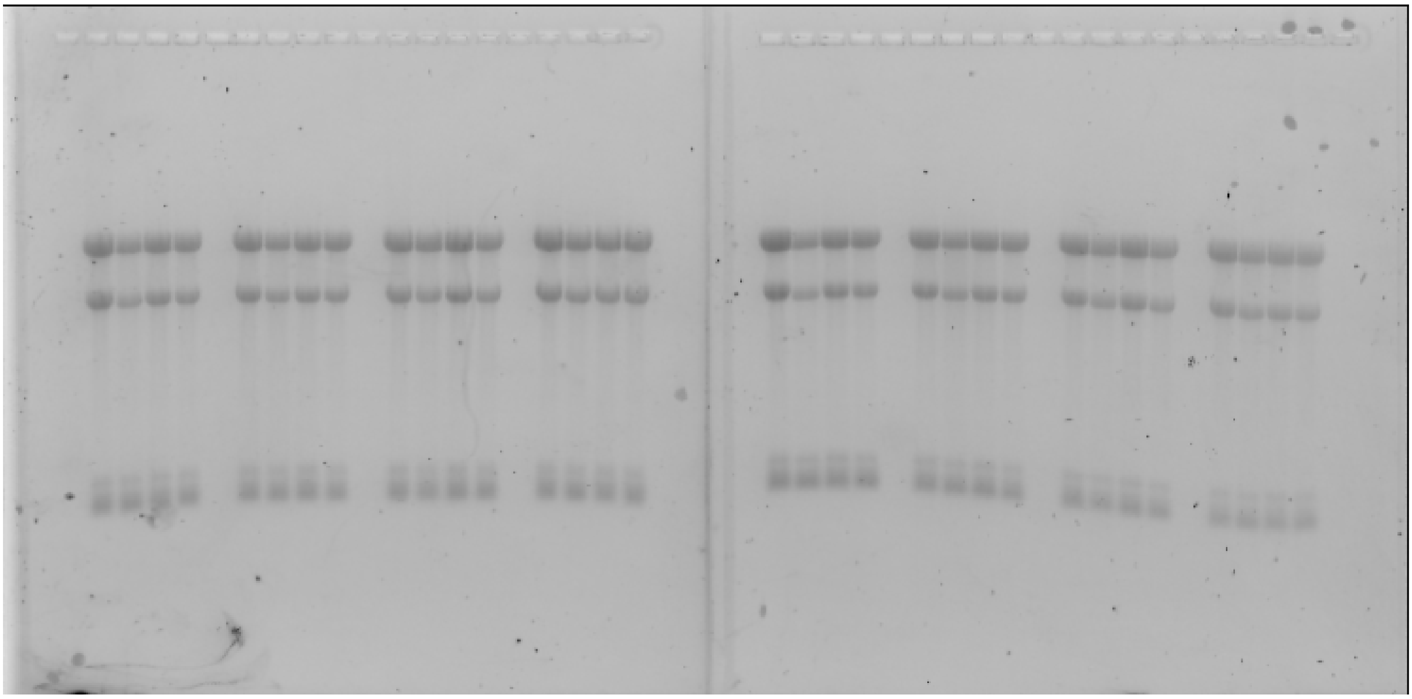

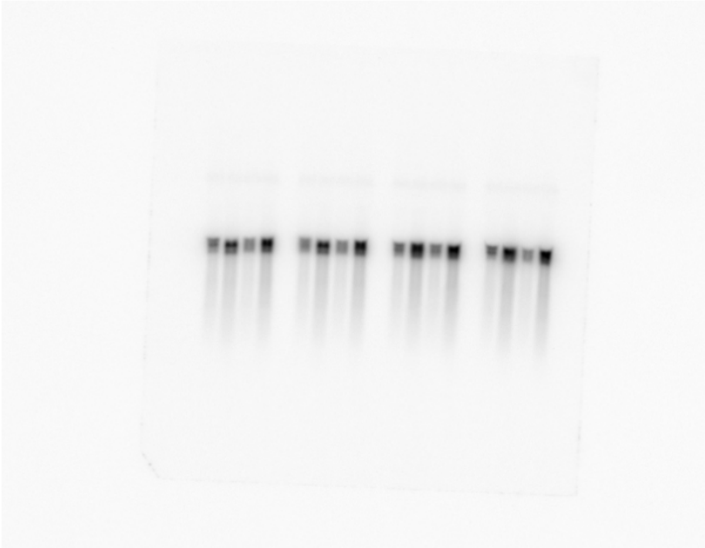

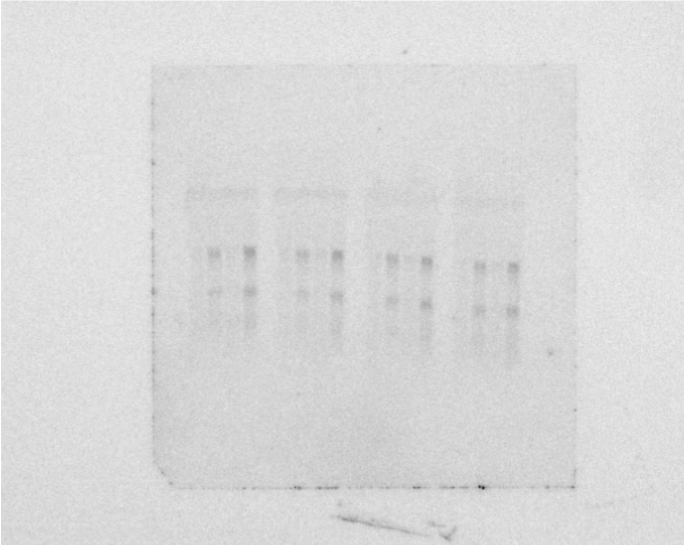

Northern blot from Fig. S6d. Samples were run on two replicate gels (ethidium bromide stained gels shown), blotted and probed for SFA1 (left) and SFA1 upstream antisense (right). Samples are organised as 4 replicates.
